# Structure-guided mutagenesis of Henipavirus receptor-binding proteins reveals molecular determinants of receptor usage and antibody-binding epitopes

**DOI:** 10.1128/jvi.01838-23

**Published:** 2024-03-01

**Authors:** Kasopefoluwa Y. Oguntuyo, Griffin D. Haas, Kristopher D. Azarm, Christian S. Stevens, Luca Brambilla, Shreyas S. Kowdle, Victoria A. Avanzato, Rhys Pryce, Alexander N. Freiberg, Thomas A. Bowden, Benhur Lee

**Affiliations:** 1 Department of Microbiology, Icahn School of Medicine at Mount Sinai, New York, New York, USA; 2 Division of Structural Biology, Wellcome Center for Human Genetics, University of Oxford, Oxford, United Kingdom; 3 Department of Pathology, University of Texas Medical Branch, Galveston, Texas, USA; University of Kentucky College of Medicine, Lexington, Kentucky, USA

**Keywords:** Henipavirus, Nipah virus, Hendra virus, Ghana virus, receptor-binding protein, antibody, virus entry, EFNB2/EFNB3, structural biology, molecular biology

## Abstract

Nipah virus (NiV) is a highly lethal, zoonotic Henipavirus (HNV) that causes respiratory and neurological signs and symptoms in humans. Similar to other paramyxoviruses, HNVs mediate entry into host cells through the concerted actions of two surface glycoproteins: a receptor-binding protein (RBP) that mediates attachment and a fusion glycoprotein (F) that triggers fusion in an RBP-dependent manner. NiV uses ephrin-B2 (EFNB2) and ephrin-B3 (EFNB3) as entry receptors. Ghana virus (GhV), a novel HNV identified in a Ghanaian bat, uses EFNB2 but not EFNB3. In this study, we employ a structure-informed approach to identify receptor-interfacing residues and systematically introduce GhV-RBP residues into a NiV-RBP backbone to uncover the molecular determinants of EFNB3 usage. We reveal two regions that severely impair EFNB3 binding by NiV-RBP and EFNB3-mediated entry by NiV pseudotyped viral particles. Further analyses uncovered two-point mutations (^NiV^N557S^GhV^ and ^NiV^Y581T^GhV^) pivotal for this phenotype. Moreover, we identify NiV interaction with Y120 of EFNB3 as important for the usage of this receptor. Beyond these EFNB3-related findings, we reveal two domains that restrict GhV binding of EFNB2, confirm the HNV-head as an immunodominant target for polyclonal and monoclonal antibodies, and describe putative epitopes for GhV- and NiV-specific monoclonal antibodies. Cumulatively, the work presented here generates useful reagents and tools that shed insight to residues important for NiV usage of EFNB3, reveal regions critical for GhV binding of EFNB2, and describe putative HNV antibody-binding epitopes.

**IMPORTANCE:**

Hendra virus and Nipah virus (NiV) are lethal, zoonotic Henipaviruses (HNVs) that cause respiratory and neurological clinical features in humans. Since their initial outbreaks in the 1990s, several novel HNVs have been discovered worldwide, including Ghana virus. Additionally, there is serological evidence of zoonotic transmission, lending way to concerns about future outbreaks. HNV infection of cells is mediated by the receptor-binding protein (RBP) and the Fusion protein (F). The work presented here identifies NiV RBP amino acids important for the usage of ephrin-B3 (EFNB3), a receptor highly expressed in neurons and predicted to be important for neurological clinical features caused by NiV. This study also characterizes epitopes recognized by antibodies against divergent HNV RBPs. Together, this sheds insight to amino acids critical for HNV receptor usage and antibody binding, which is valuable for future studies investigating determinants of viral pathogenesis and developing antibody therapies.

## INTRODUCTION

In the 1990s, Hendra virus (HeV) and Nipah virus (NiV) were the zoonotic agents behind highly lethal outbreaks in Australia, Malaysia, and Singapore (1–3). These two viruses became the prototypic members of the Henipavirus (HNV) genus, a member of the paramyxovirus family, and have caused over 20 human outbreaks with a case fatality rate approaching 60% (4). Human cases of NiV infection manifest clinically with respiratory and neurological signs and symptoms (1). Of these, the development of neurological signs and symptoms is most strongly associated with mortality (5, 6). Moreover, there is post-mortem evidence of NiV infection of neurons, the presence of the virus in cerebrospinal fluids, and long-term neurological sequelae after acute NiV infection (5, 7, 8).

Since the original outbreaks, a new HeV genotype (HeV-g2) (9, 10) and several new members have been added to the HNV genus, including Mojiang virus (MojV) from Rattus flavipectus in China, Cedar virus (CedV) from Pteropus bats in Australia, and Ghana virus (GhV) from Eidolon helvum bats in Ghana (11–13). More recently, Langya virus (LangV), a novel HNV with a presumed Soricidae shrew natural host, was identified as the putative cause of a febrile illness in China between 2019 and 2021 (14). The discovery of novel HNVs, recent outbreaks in China and India, and serological evidence of spillover events in Cameroon lends way to concerns about future outbreaks and highlights the need for a better understanding of receptor usage and its role in viral pathogenicity (14, 15).

HNVs encode two surface glycoproteins, the receptor-binding protein (RBP) and the Fusion protein (F), that facilitate entry. The tetrameric RBP has a head composed of a six-bladed beta-propeller structure that mediates binding and a stalk that mediates dimerization and tetramerization through cysteine bridges (16, 17). The engagement of the RBP with a receptor induces conformational changes that trigger activation of the trimeric F, which undergoes changes to form a pre-hairpin intermediate that exposes the fusion peptide at the viral membrane distal region and subsequently creates a six-helix bundle to form a fusion pore.

NiV and HeV have been shown to use Ephrin-B2 (EFNB2) and Ephrin-B3 (EFNB3), while GhV only utilizes EFNB2 (18–21). CedV was recently characterized to use EFNB2, Ephrin-B1 (EFNB1), and select Ephrin-As (22, 23). Meanwhile, receptors have not yet been identified for MojV, LangV, or more recently discovered HNV (11, 14, 24–27). Previous groups have leveraged alanine mutagenesis or reciprocally exchanged residues to identify amino acids on both the RBP and receptor side important for receptor usage, particularly for EFNB2 (28–30). Notably, some approaches identified HeV-S507 as important for EFNB3 binding (31), NiV-Q533 as a residue important for EFNB2 and EFNB3 binding (29), and NiV-QY388-89 as a region important for stabilizing the interaction of HNV-RBP with EFNB2 (21). On the receptor side, structural and functional studies have identified that residues within the GH loop of the ephrin-Bs (EFNBs) snugly fit into pickets lined by HNV residues (23, 28, 32–34). A recent study leveraged this GH loop to generate decoy EFNB2 mutants that bind to HNVs but not cognate Eph receptors (35).

Given the neurotropism of NiV infections and its association with mortality, it is hypothesized that the usage of EFNB3, a receptor highly expressed in the central nervous system, may be implicated in the pathogenicity of NiV. However, prior RBP and receptor studies primarily focus on HNV engagement with EFNB2, a receptor broadly expressed across several tissues (36, 37). To study the molecular determinants of EFNB3 usage, we leverage NiV (an EFNB2 and EFNB3 using HNV) and GhV (an EFNB2 using, but EFNB3-blind HNV) as HNV-RBPs with distinct receptor usage to characterize regions necessary to impair EFNB3 usage by NiV. Through systematic, structure-informed mutagenesis, we determine the minimal residues essential to prevent EFNB3 usage by NiV and reveal the distinct accommodations made by NiV to interact with EFNB3. With mutants generated in this study, we also identified regions of GhV that play an important role in EFNB2 usage and identify putative-binding epitopes for novel GhV-specific monoclonal antibodies.

## RESULTS

### The head domain of HNVs is a critical determinant of EFNB3 binding

**Fig 1:**
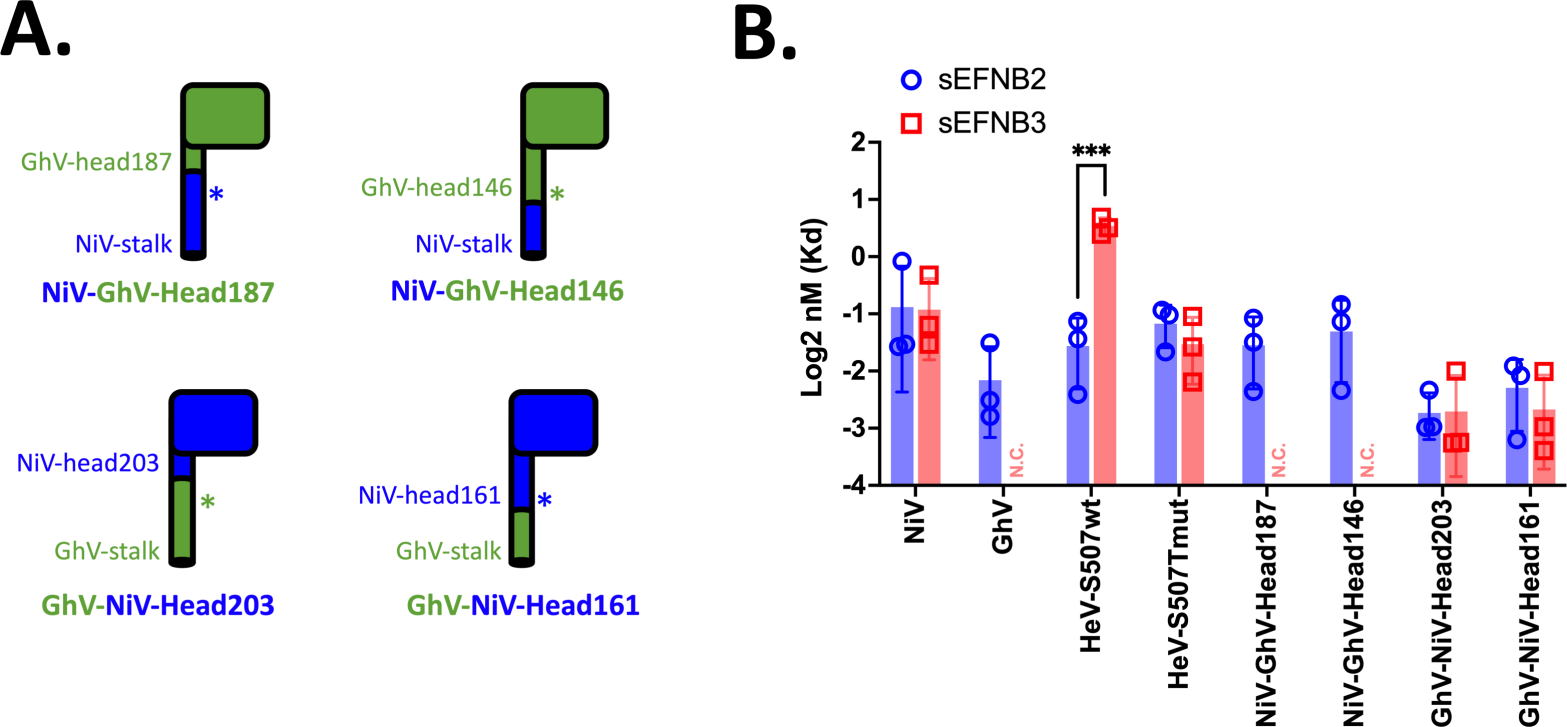
EFNB3 binding localizes to the head domain of HNV RBP. (**A**) Schematic of head-stalk chimeras generated. Constructs presented on top bear a NiV-RBP stalk fused to the GhV head domain at the indicated amino acid position. The two constructs below bear the GhV-RBP stalk fused to the NiV head domain. The asterisks represent the cysteine residues important for HNV tetramerization. Both constructs on the right have a shorter stalk and bear the cysteine residues from the transplanted head. (**B**) Binding of HNV RBPs to soluble EFNB2 and soluble EFNB3. Flow cytometry was performed by transfecting 293T cells with the indicated HA-tagged HNV RBP then stained with a serial dilution of Fc-tagged receptor. This is further described in the Materials and methods. Full-binding curves are presented in Fig. S1. For each point on the curve, the background was first subtracted, then normalized to anti-HA. All data points were further normalized to the highest concentration of soluble receptor tested (50 nM). Data were analyzed using a non-linear regression with a saturation-binding model with one site. Dissociation constants (K_d_) presented were calculated from each of three independent, biological replicates. N.C. indicates not calculated due to the absence of binding by soluble receptors. An unpaired *t* test was performed to calculate statistical significance for all groups with calculated K_d_s for EFNB2 and EFNB3 (***P < 0.0002).

To begin elucidating regions that distinguish NiV and GhV usage of EFNB2 and EFNB3, we generated head-stalk chimeras that exchanged the head domain between two RBPs. Others have shown that transfer of the NiV-head to Newcastle Disease Virus (NDV) confers EFNB2 binding and usage to NDV (38, 39). However, we first wanted to assess whether the transfer of the GhV-head to the NiV-stalk ablates NiV binding of EFNB3 while retaining EFNB2 binding. Using a flow cytometry-based-binding assay with soluble EFNB2 (sEFNB2) or soluble EFNB3 (sEFNB3), we observe that transfer of the GhV head domain with truncations at either the base of the globular head (NiV-GhV-Head187) or the stalk (NiV-GhV-Head146) rendered the chimeras unable to bind EFNB3 while preserving EFNB2 binding. The converse mutations in the GhV-backbone (GhV-NiV-Head161, truncated within the stalk, and GhV-NiV-Head-203, truncated at the base of the globular head) conferred EFNB3 binding while maintaining EFNB2 binding (Fig. 1; Fig. S1). As internal controls for the sensitivity of the flow cytometry assay, we included two HeV-RBP constructs that have been previously described to impart differences in EFNB3 binding. Consistent with the literature, we observe statistically significant reduction in sEFNB3 binding by HeV-RBP bearing a serine at amino acid position 507 (S507; Kd = 1.45 nM for sEFNB3 vs 0.34 nM for sEFNB2), but equivalent EFNB2 and EFNB3 binding is restored upon the introduction of a threonine at that position (S507T) (31).

To assess the entry of each chimera into relevant cell lines, we generated VSVΔG-RLuc HNV pseudotyped particles (HNVpp) bearing each of the RBPs and homotypic or heterotypic matched F glycoproteins. Consistent with our binding results, we observed that the GhV-NiV-Head chimeras were able to enter both ephrin-B2 and ephrin-B3 expressing CHO cells (Cho-B2 and Cho-B3). The NiV-GhV-Head chimeras showed no entry into Cho-B3 cells and defective entry into Cho-B2 cells. However, modest levels of entry were observed in the highly permissive U87 cells for three of four NiV-RBP chimeras and F combinations tested (Fig. S2A). For the NiV-GhV-head chimeras, we hypothesize that this observed reduced entry may be due to decreased incorporation into the VSV∆G particles (Fig. S2B) or restricted compatibility of the HNV-RBP chimera stalks with the paired F glycoprotein.

### Systematic, structure-informed mutagenesis reveals residues important for EFNB3 binding and usage

**Fig 2:**
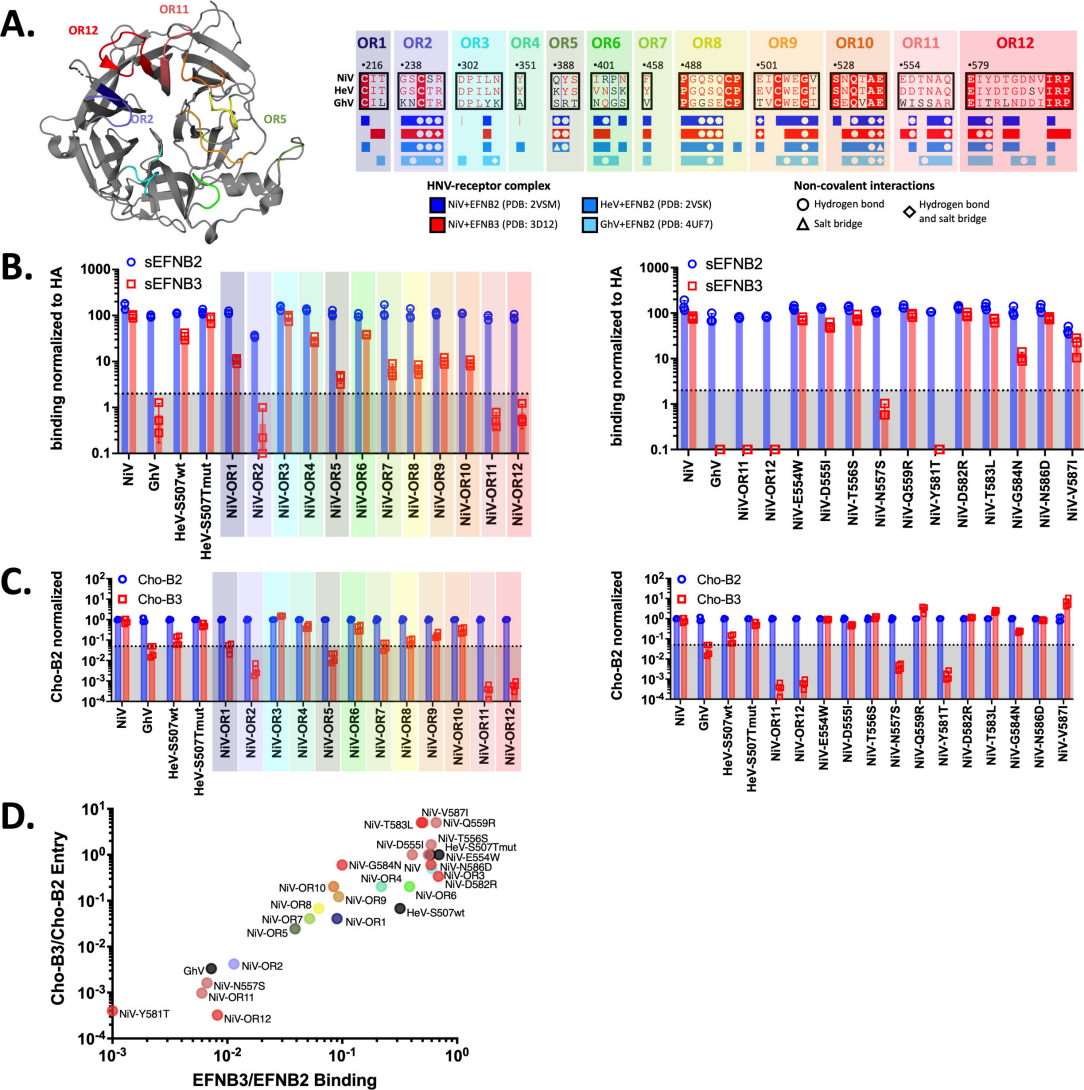
Systematic, structure-guided mutations identify N557 and Y581 as essential for EFNB3 binding and usage. (**A**) Regions occluded by NiV-RBP interaction with EFNB2 displayed on NiV-RBP structure (left) and HNV-RBP alignment (right). Multalin and ESPript were used to identify OR 1–12 between the NiV-RBP and EFNB2 and visualized on the NiV-RBP structure. (**B**) Binding of sEFNB2 and sEFNB3 to NiV-OR1-12 (left) and selected point mutants (right). Binding was assessed by flow cytometry as described in the Materials and methods. Presented are data of soluble receptor binding at 2 nM (10 and 0.4 nM in Fig. S3). The dotted line at 2 indicates an approximately 50× reduction in receptor binding relative to wild-type NiV. Presented are the results from three independent, biological replicates. (**C**) Entry into Cho cells stably expressing EFNB2 (Cho-B2) or EFNB3 (Cho-B3) by NiV-OR1-12 (left) and selected point mutants (right). HNVpps bearing the respective RBPs with homotypic F glycoproteins were prepared and tittered on Cho-B2 and Cho-B3 cells as described in the Materials and methods. Data presented are a single dilution and were normalized to average cho-B2 entry for each sample. Presented are the results of two independent, biological replicates performed in technical replicates (*n* = 4). The full titers are presented in Fig. S5. The highlighted ORs in Fig. 2B and C (left) follow the same coloring scheme presented in Fig. 2A (right). Entry for NiV, GhV, HeV wt +mut, NiV-OR11, and NiV-OR12 repeated in the figure on the right for ease of data interpretation. (**D**) Binding and entry ratios for EFNB3 to EFNB2. The ratio of normalized soluble EFNB3:EFNB2 binding values at 2 nM was calculated from Fig. 2B and presented on the X-axis. NiV-Y581T was given a binding ratio of 0.001 due to having a negative value after subtracting the geometric mean fluorescent intensity (GMFI) background. The ratio of EFNB3:EFNB2 titers was calculated from the average titers on Cho-B2 and Cho-B3 cells presented in Fig. S5. Each point is colored based on the coloring scheme presented in Fig. 2A right. Control samples (GhV, NiV, and HeV) have black symbols.

Once established that EFNB3 usage could be ablated in NiV or conferred to GhV by the transfer of the head domain, we leveraged GhV-RBP as an EFNB3-blind HNV to further interrogate specific regions necessary to impair EFNB3 usage by NiV. Using a tool to explore molecular interfaces (PDBePISA) (40) with extant crystal structures of NiV, HeV, and GhV RBP in complex with EFNB2 (21, 32), we identified residues occluded upon the interaction of each RBP with EFNB2. These analyses were also extended to NiV in complex with EFNB3 (41). The results were then visualized on the NiV-EFNB2 structure and annotated onto an alignment of HNV sequences and divided into 12 distinct regions that were subsequently named occluded regions (ORs). Interestingly, NiV, HeV, and GhV RPBs share nearly identical interaction profiles for residues occluded upon interaction with EFNB2 or EFNB3 (Fig. 2A).

We subsequently introduced the GhV-RBP residues from individual 12 ORs into a NiV backbone and assessed the effect on binding with a flow cytometry assay. Many regions impair EFNB3 binding, suggesting that several amino acids across the receptor-binding pocket support EFNB3 binding. However, OR2, OR5, OR11, and OR12 have the most pronounced effect with approximately 20× to 100× reductions in EFNB3 binding relative to EFNB2 binding (Fig. 2B, left). Interestingly, OR2 also impairs EFNB2 binding. This may be due to the introduction of an N-linked glycan from GhV’s OR2 into the NiV backbone, which is supported by the presence of a higher molecular weight RBP glycoprotein on western blot (Fig. S4). Given OR11 and OR12 displayed the largest reductions in EFNB3 binding, we subsequently introduced individual point mutations from these regions into the NiV-backbone. Here, we observe severely restricted EFNB3 binding by mutations to N557 and Y581. Since we observe only modest decreases in EFNB3 binding by other point mutations, this finding suggests N557S and Y581T are implicated in the phenotype observed with OR11 and OR12, respectively (Fig. 2B, right). Binding was also assessed at 10 and 0.4 nM for all constructs. When binding to excess sEFNB3 (10 nM), NiV-OR11, OR12, N557S, and Y581T still displayed drastic impairments in binding (Fig. S3).

To assess the functional impact of these regional and point mutations on entry into Cho-B2 and Cho-B3 cells, we generated HNVpp bearing each RBP construct and homotypically matched F glycoproteins. As expected from the literature, NiV showed nearly equivalent entry into both cell lines, HeV-S507T had similar entry into both cell lines, HeV-S507 displayed reduced entry into Cho-B3 cells relative to Cho-B2 (31), and GhV displayed no entry into Cho-B3 cell lines (21) (Fig. 2C). Titers were calculated for these control constructs, and our chimeric constructs and data were normalized to Cho-B2 entry for each individual construct (Fig. S5; Table S1). We observe the greatest reductions in Cho-B3 entry for NiV-OR2, OR5, OR11, and OR12 mutants. Specifically, NiV-OR11 and OR12 display over 3,000× and 1,500× reductions in EFNB3-mediated entry when normalized to Cho-B2, respectively. Within these regions, point mutants N557S and Y581T display approximately 250× and 500× reductions in EFNB3-mediated entry. Although each point mutant accounts for substantial reductions in EFNB3 usage, these findings suggest that mutations from each region work synergistically to have the greatest impact on EFNB3 usage. Moreover, select point mutations, such as Q559R, T583L, and V587I, modestly enhance Cho-B3 entry approximately 2× to 4× when normalized to Cho-B2 entry (Fig. 2C, right). Interestingly, these constructs also display reduced EFNB3 binding (Fig. 2B, right) but have significantly reduced titers when compared to wild-type (WT) NiV (Table S1). By taking a ratio of EFNB3 to EFNB2 binding and Cho-B3 to Cho-B2 entry data, we summarize our findings for over 25 constructs. Here, it can be clearly appreciated that NiV-OR11, OR12, N557S, and Y581T have the greatest impact on EFNB3 binding and Cho-B3 entry (Fig. 2D).

### NiV ORs do not confer EFNB3 usage to GhV and shed insight to GhV EFNB2 usage

**Fig 3:**
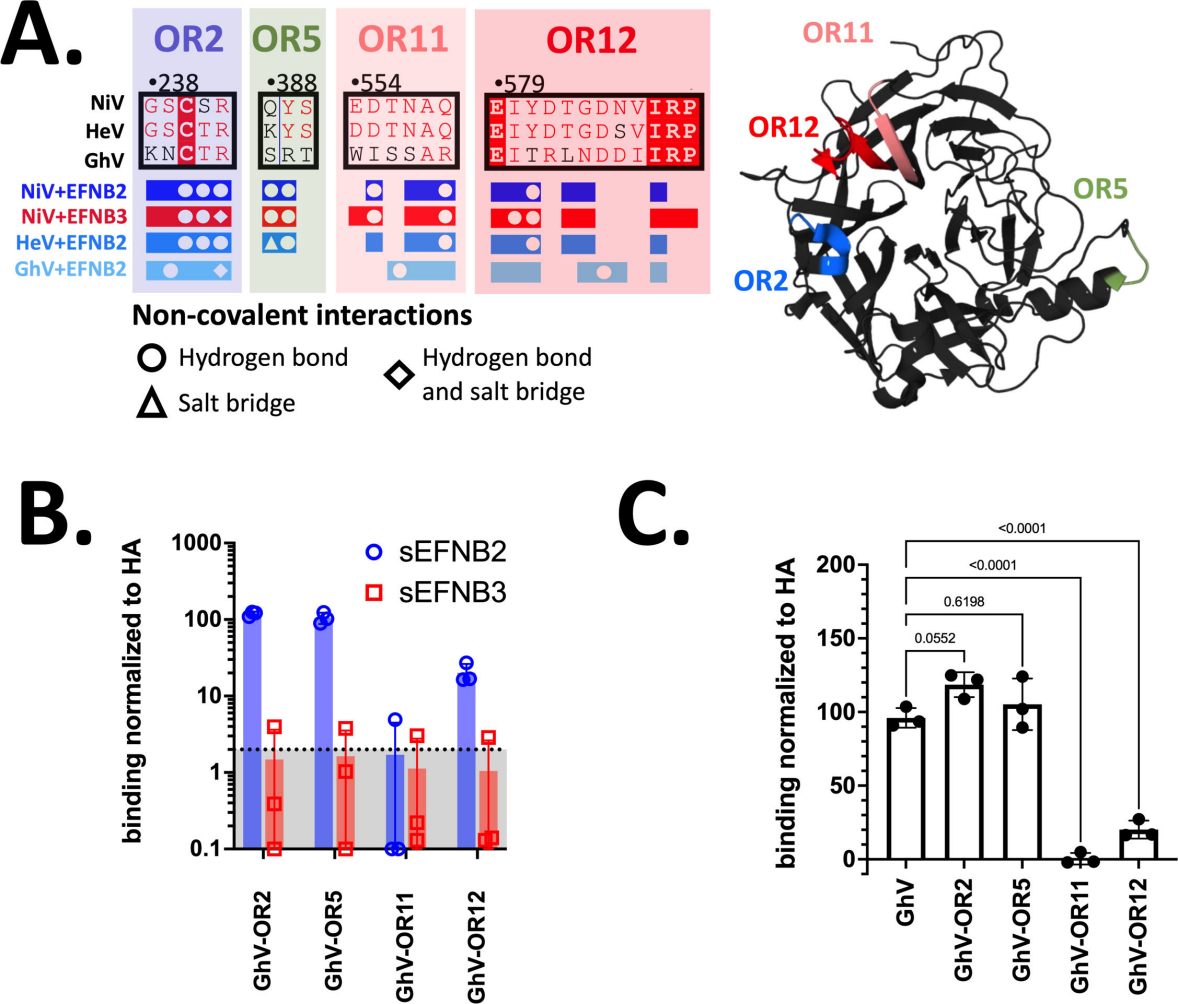
Introduction of NiV RBP residues into a GhV backbone does not confer EFNB3 binding. (**A**) Schematic of ORs. Fig. 2A was adapted to present ORs of interest in this figure. Visualization of the localization of each OR within the GhV-RBP structure. Pymol was used to map the location of OR2, 5, 11, and 12 on the GhV-G structure (PDB:4UF7). Each region is colored the same as in Fig. 2A, right. (**B**) Binding of GhV-OR mutants to EFNB2 and EFNB3. The binding of receptors at 2 nM is presented here and was performed as described in Fig. 2A. These are the results from three independent, biological replicates. Binding at 10 and 0.4 nM can be found in Fig. S6. (**C**) Binding of GhV-OR mutants to EFNB2. Presented are the data from Fig. 3B, but only EFNB2 binding with the addition of GhV-WT-RBP. Statistical significance was assessed with a one-way ANOVA with Dunnet’s correction for multiple comparisons. *P* values are presented atop each comparison.

We next sought to assess if any of these domains are sufficient to confer EFNB3 binding to GhV-RBP by transferring the ORs most implicated in NiV binding of EFNB3. These regions (OR2, 5, 11, and 12) were mapped onto the structure of GhV (Fig. 3A, right). In binding studies, all the chimeric GhV constructs (GhV-OR2, OR5, OR11, and OR12) were still unable to bind EFNB3 at soluble protein concentrations of 2 (Fig. 3B) and 10 nM (Fig. S6A). Additionally, we transferred several domains from NiV-RBP to generate GhV-OR+ (bearing NiV ORs 2 + 5 + 11 + 12) or GhV-OR++ (bearing NiV ORs 1 + 2 + 5 + 7 + 8 + 11 + 12), but these chimeric constructs expressed poorly at the cell surface and did not bind either EFNB2 or EFNB3 at 10 nM (Fig. S6D).

Further analyses of individual OR mutants in the GhV backbone revealed interesting trends with regard to EFNB2 binding. The removal of the N-linked glycan at OR2 trends toward significantly increased EFNB2 binding at 2 nM (Fig. 3C) and has statistically significant EFNB2 binding at 10 and 0.4 nM (Fig. S6B). Interestingly, GhV-OR11 and GhV-OR12 mutants displayed significant reductions in EFNB2 binding at 2 nM (Fig. 3C) and other concentrations tested (Fig. S6B). To assess whether this loss of binding is due to disruption of normal GhV conformations, we utilized two RBD-specific, anti-GhV mouse monoclonal antibodies (1E10 and 1G1) to assess cell surface expression of the GhV chimera. Both antibodies bind GhV-OR2, OR11, and OR12. However, these antibodies fail to bind GhV-OR5, suggesting they share an overlapping binding epitope (Fig. S6C).

### Accommodation of EFNB3 Y120 is important for EFNB3 binding and usage

**Fig 4:**
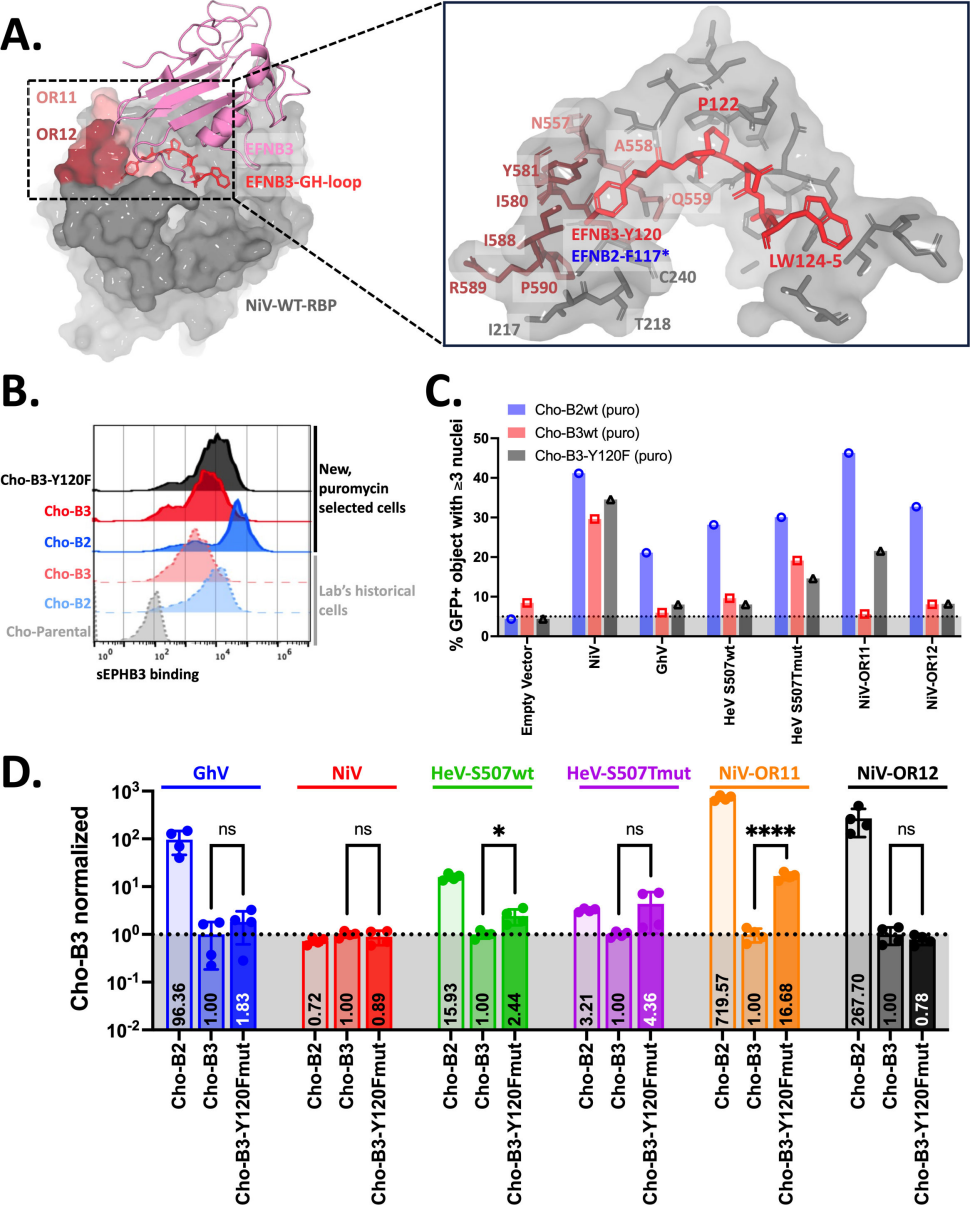
NiV interaction with EFNB3 residue Y120 is important for EFNB3 usage. (**A**) EFNB3-Y120 is primarily enclosed by NiV-OR11 and OR12 residues. Pymol was used to generate complexes of NiV-EFNB3 (PDB:3D12). EFNB3 in complex with NiV-WT RBP (left) and stick renderings of EFNB3 GH loop residues 120–125 and the NiV-binding pocket (right). The asterisk indicates the EFNB2 residue at the same position. (**B**) Cell surface expression of WT EFNB2, WT EFNB3, and mutant EFNB3-Y120F on new, puromycin-selected cell lines was compared to our lab’s historical cell lines. Cells were stained using a fivefold serial dilution of soluble EPHB3 (1,210–0.4 nM). Representative histograms are shown for binding to 242 nM sEPHB3. The remaining dilutions tested are quantified and presented in Fig. S8. (**C**) NiV-OR11 forms syncytia on EFNB3-Y120F mutant expressing cells. This method to screen for syncytia formation is described in the Materials and methods and was validated in Fig. S9. (**D**) HNVpp entry into EFNB3-Y120F mutant cell lines. The respective cell lines were infected with the HNVpp, and the data shown are from the dilutions indicated on the left side. Data were normalized to average Cho-B3 relative light units (RLUs), and presented are the results of two independent, biological replicates performed in technical duplicates. Statistics shown are an unpaired *t* test (ns = not significant, *P < 0.03, and ****P < 0.0001). Raw RLUs for each biological replicate are presented in Fig. S8.

Next, we sought to assess why NiV-OR11 and OR12 cannot effectively use EFNB3 while still retaining EFNB2 usage. EFNB2 and EFNB3 are highly conserved across species and in HNV-interacting residues (Fig. S7). However, within HNV-interacting residues proximal to OR11 and OR12, EFNB2 bears a phenylalanine at position 117 (F117), and EFNB3 bears a tyrosine at position 120 (Y120). Despite only differing by the addition of a single hydroxyl group found in tyrosine, both residues are buried within a pocket formed at the interface of both OR11 and OR12 (Fig. 4A). As a result, we hypothesized that NiV-OR11 and NiV-OR12 chimeras cannot effectively use EFNB3 due to an inability to accommodate Y120. To address this question, we generated cell lines stably expressing WT EFNB2, WT EFNB3, or mutant EFNB3 bearing the EFNB2 phenylalanine at position 120 (EFNB3-Y120F). These cell lines were validated as expressing their respective receptor by using flow cytometry to detect the binding of EPHB3, a cognate receptor previously reported to bind EFNB2 and EFNB3 (42) (Fig. 4B).

To further validate these cell lines, we developed a green fluorescent protein (GFP)-based syncytia assay to quantitatively evaluate syncytia formation across several cell lines as a complementary approach to assess for receptor usage prior to producing HNVpp. For this cell-cell fusion assay, Cho-B2 or Cho-B3 cells were transfected with HNV glycoproteins and an eGFP fluorescent protein, then imaged approximately 24 h post transfection to capture cells/syncytia as indicated by the GFP+ fluorescence and nuclei within cells/syncytia as indicated by Hoechst staining. Subsequently, we used CellProfiler (43, 44), an imaging processing tool, to automate cell/syncytia identification by associating GFP+ cells with nuclei within individual GFP+ cells. For this assay, we considered syncytia to be a GFP+ object with three or more nuclei given. This assay is further described in the Materials and methods and was validated extensively for both our historical and newly generated Cho-B2 and Cho-B3 cells (Fig. S9). While this quantitative, syncytia assay is a measure of cell-cell fusion and not direct viral entry, it has a positive correlation to the HNVpp model of viral entry (Fig. S9G). In the new, puromycin-selected cell lines, NiV displayed similar entry into Cho-B2/Cho-B3 cells, and GhV displayed entry only into Cho-B2 cells. Meanwhile, in the puromycin-selected, WT Cho-B3 cells, GhV, NiV-OR11, and NiV-OR12 showed background levels of syncytia formation. Notably, with NiV-OR11, but not NiV-OR12, syncytia formation was restored in Cho-B3-Y120F cells (Fig. 4C).

**Fig 5:**
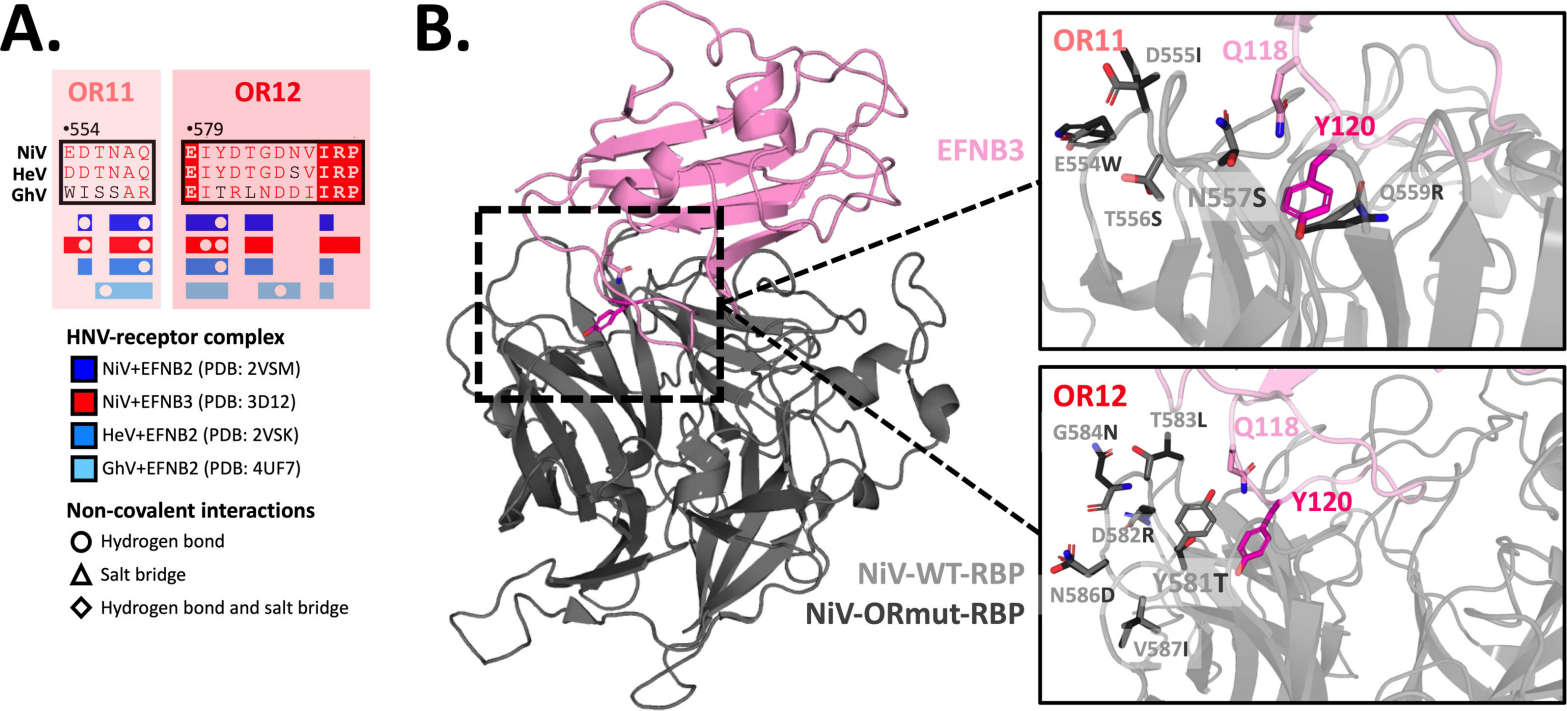
Structural rationale for N557S and Y581T disruption of EFNB3 binding and usage. (**A**) OR11 and OR12 residues occluded by interaction with cognate receptors. This annotation was generated using Multalin, ESPript, and PDBePISA as described in Fig. 2. (**B**) Superimpositions of WT NiV-RBP and NiV-OR11 or NiV-OR12 structures. Structures bearing the mutant residues at NiV-OR11 and NiV-OR were generated in COOT. These were then aligned onto NiV-wt-RBP in complex with EFNB3 (PDB: 3D12) in pymol. The full structure is presented on the left with EFNB3 presented in pink and WT-RBP in gray. The right panel shows insets zoomed into a region of the interaction with OR11 (top right) or OR12 (bottom right) with WT residues presented in light gray and mutant residues presented in dark gray.

We subsequently produced HNVpps and assessed entry into all three puro-selected cell lines. With the newly generated cell lines, control HNVpp continued to display entry in line with previous observations as GhVpp effectively infected Cho-B2 cells and NiVpp effectively infected both Cho-B2 and Cho-B3 cells. Additionally, consistent with our syncytia assay results, NiV-OR11pp, but not NiV-OR12pp, display a statistically significant increase in entry into Cho-B3-Y120F over WT Cho-B3 cells (Fig. 4D). These results emphasize that OR11 plays a role in the accommodation of the hydroxyl-bearing tyrosine residue within EFNB3. Interestingly, when this syncytia assay was expanded to include individual point mutations from OR11 and OR12, we did not observe any Cho-B3-Y120F syncytial phenotype as dramatic as that from NiV-OR11, implicating multiple amino acids as important for this dynamic accommodation (Fig. S9F). Since NiV-OR12 usage of EFNB3 was not rescued with the Cho-B3-Y120F, we interrogated the structure and identified that Y581T removes a tyrosine from NiV, thus preventing pi-stacking necessary to interact with Y120 of EFNB3 (Fig. 5B).

### Repurposing head-stalk chimeras and OR mutants to map antibody-binding epitopes

**Fig 6:**
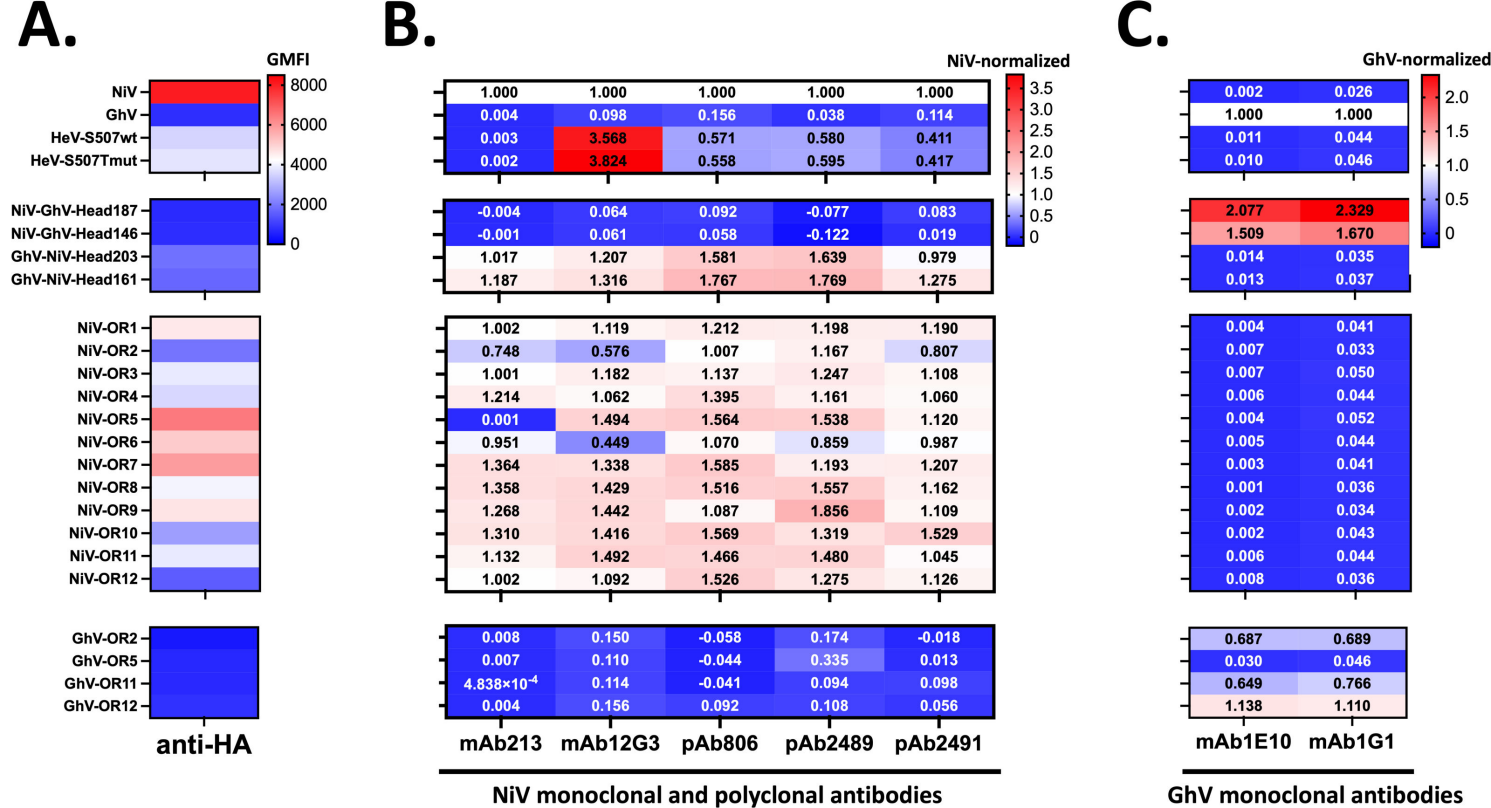
Head-stalk chimeras reveal the immunodominance of HNV-heads, and OR mutants identify putative epitopes for HNV antibodies. (**A**) Cell surface expression of constructs as indicated by anti-HA. Flow cytometry was performed as described in the Materials and methods. Briefly, cells were transfected with the indicated HNV RBP, then stained 2 days post transfection. Presented are the background subtracted GMFI from one experiment. (**B**) Binding of constructs to NiV monoclonal and polyclonal antibodies. Flow cytometry was performed as described above, and background-subtracted GMFI was first normalized to anti-HA GMFI to account for variations in cell surface expression. These values were further normalized to NiV. Presented are the results from one experiment. (**C**) Binding of constructs to GhV monoclonal antibodies. Flow cytometry and analyses were performed as described in panel B of this figure with one exception. For this panel, HA-normalized binding was subsequently normalized to GhV.

Inspired by the findings that OR5 may be the binding epitope for two GhV-RBP-specific monoclonal antibodies (Fig. S6C), we repurposed 20 of the chimeras generated in this study to map epitopes for polyclonal and monoclonal antibodies (pAbs and mAbs, respectively) from our lab. Using the NiV and GhV head-stalk chimeras, we note that all antibodies tested bind to their respective head domains (Fig. 6). We further utilized our NiV and GhV OR mutants to epitope map extant mAbs in our collection as well as GhV-specific mAbs that have not been previously characterized. Here, utilizing mAb213, we identify OR5 as a binding epitope, which is consistent with a previous publication that identified Q388R as an escape mutation that developed in live virus escape studies (45). Additionally, mAb12G3, an antibody originally generated against HeV, shows greater than threefold higher binding of HeV compared to NiV. NiV-OR2 and NiV-OR6 display about 50% decreased binding to mAb12G3, suggesting these may be peripheral epitopes of the mAb12G3-binding footprint. Lastly, mAbs 1E10 and 1G1 bind to GhV OR2, OR11, and OR12 mutants. However, both antibodies display decreased binding to GhV-OR5, suggesting that this region may represent a binding epitope shared by both antibodies (Fig. 6).

## DISCUSSION

In the work presented here, we leverage extant structures of NiV and GhV RBPs to interrogate regions important for EFNB3 usage, a receptor hypothesized to be implicated in the neurotropism and mortality seen with NiV. Through systematic regional mutations, we identify OR11 and OR12 as important domains for EFNB3 binding and usage. Through point mutations from these regions, we characterize N557 and Y581 as residues driving this phenotype (Fig. 2). Moreover, BSL4 experiments with recombinant NiV-bearing N557S, Y581T, and OR5 mutations continue to display severely impaired EFNB3 usage (Fig. S10) and may prove to be important tools to assess the role of EFNB3 in the neuropathogenicity of NiV.

We further show that transfer of single ORs did not confer EFNB3 usage to GhV, suggesting that multiple regions or acquisition of non-contiguous mutations may be necessary to enable GhV-RBP binding and usage of EFNB3 (Fig. 3B). Interestingly, CedV, an HNV with promiscuous binding to EFNB2, EFNB1, and select ephrin-As, may require fewer mutations to enable EFNB3 usage (22, 23). In fact, Laing et al. explicitly highlight that CedV appears to lack the ability to pi-stack with Y120 of EFNB3 due to the presence of N602 in place of Y581 from NiV (22). This is supported by our data demonstrating Y581T ablates binding and usage of EFNB3 (Fig. 2B and C), suggesting a shared mechanism by which N602 from CedV and T590 from GhV, both amino acids with polar, neutral side chains, are unable to use EFNB3.

To investigate putative mechanisms for the selective impairment of EFNB3 usage relative to EFNB2, we turned to the receptor side and noted several amino acids buried within binding pockets created by HNV-RBPs. The binding pockets for the Leu-Trp (LW) residues in the GH loop of EFNB2 and EFNB3 are fully formed prior to the engagement with the NiV-RBP and have been functionally shown to be critical for usage of EFNB2 (20). However, structural studies suggest that the unbound NiV-RBP appears to make conformational changes to accommodate the tyrosine at amino acid position 120 (Y120) of EFNB3 (41). This residue is buried within a pocket created at the interface of OR11 and OR12. Interestingly, OR12 contains NiV residues 579–590, which were previously described in structural studies to represent a dynamic region that undergoes conformational changes upon EFNB2 engagement (46). Notably, when aligned with EFNB3, EFNB2 bears phenylalanine (F) at this position, so we hypothesized that OR11 and OR12 are unable to bind and use EFNB3 due to an inability to accommodate the hydroxyl group found in tyrosine (Fig. 4A; Fig. S7). We explored the role of EFNB3-Y120 by exchanging this residue with the F found in EFNB2 at this position to generate the mutant EFNB3-Y120F and assessed receptor usage with a novel GFP-based syncytia assay and our standard HNVpp entry assay. Here, in partial support of our hypothesis, we observe that NiV-OR11, but not NiV-OR12, usage of EFNB3-Y120F is partially rescued relative to WT EFNB3 (Fig. 4). While NiV-OR12 usage of EFNB3 is not recovered by the introduction of the Y120F mutation, we hypothesize that it is likely due to an inability for the Y581T mutation to pi-stack with Y120 of EFNB3 consistent with our data with this point mutation and predictions from the literature (22) (Fig. 5B).

From these functional studies, we also made additional observations regarding EFNB2 and EFNB3 receptor usage. For GhV, the introduction of NiV-residues from OR11 and OR12 significantly impaired EFNB2 binding at 10, 2 and 0.4 nM despite being cell surface expressed and conformationally intact as indicated by an HA tag and GhV-specific monoclonal antibodies (Fig. 3; Fig. S6). Though the GhV-RBP structure currently lacks resolution of the C-terminal tail, it is predicted to be directed up toward the interface of the beta sheets containing OR11 and OR12 (21). Given the location, we hypothesize that GhV-OR11 and OR12 mutants may interfere with the orientation of the C-terminal tail and subsequently impair GhV-RBP from maintaining a conformation necessary for proper receptor engagement. Interestingly, the NDV Ulster strain has an RBP/HNV C-terminal extension that is involved in viral entry by mediating dimerization and inhibition of sialic acid binding (47). Similarly, the truncation of the C-terminal tail of GhV-RBP has been reported to reduce syncytia formation, suggesting a downstream role in fusion activation (21). However, the mechanism driving this phenotype has not been characterized, and our results suggest a preceding, putative role of the RBP C-terminal tail in receptor binding.

For NiV, we note that NiV point mutants Q559R, T583L, and V587I show modestly decreased EFNB3 binding yet display enhanced Cho-B3 entry when normalized to Cho-B2 (Fig. 2; Table S1). This discrepancy between binding and entry indicates changes in EFNB3 fusion dynamics with these point mutations. Previous studies have shown subtle conformational changes induced within NiV-RBP upon engagement with EFNB2 through hydrogen-deuterium exchange studies and enhanced binding of distinct regions by two monoclonal antibodies (48–50). While not mechanistically resolved in this work, our results suggest distinct conformational changes induced by NiV binding of EFNB3 compared to EFNB2. This is consistent with our lab’s previous observation that mAb45 binding to NiV-RBP is enhanced in the presence of EFNB2, but not EFNB3 (50). Additionally, a recent structural study highlights a distinct, thumb-like domain that HPIV3-RBP utilizes to engage its fusion glycoprotein prior to fusion triggering and identifies comparable regions within other paramyxovirus that may play similar roles (51). This region is predicted to be in NiV-RBP amino acids 418–425, which is outside the receptor-binding pocket for NiV but may be a region that undergoes post-receptor binding conformational changes shared by both EFNB2 and EFNB3.

Lastly, inspired by our fortuitous mapping of putative-binding epitopes of two GhV monoclonal antibodies (Fig. S6), we further leveraged constructs generated in this study to assess binding epitopes for three polyclonal antibodies and four monoclonal antibodies. Here, we observe that the globular head domain is immunodominant as the NiV-head-stalk chimeras (bearing the NiV-stalk and GhV-head) lose reactivity to the NiV-derived polyclonal and monoclonal antibodies (Fig. 6). Interestingly, although the polyclonal antibodies 2,489 and 2,491 were generated by immunizing rabbits with DNA-encoding full-length RBP, none of the antibodies appear to be stalk reactive. These findings suggest that the head domain of HNVs is immunodominant, or the globular head may shield the stalk from recognition by reactive antibodies. A recent study shows that depletion of head-reactive antibodies from sera from African Green Monkeys immunized with soluble tetrameric NiV-RBP ablates *in vitro* neutralization potential (52), which supports our findings that polyclonal antibodies are directed largely toward the head domain. Unsurprisingly, the introduction of no single OR impairs polyclonal antibody binding.

Moreover, we identify putative-binding epitopes for NiV monoclonal antibodies (mAbs 213 and 12G3) and GhV monoclonal antibodies (mAbs 1E10 and 1G1; Fig. 6). These putative epitopes are supported by the previous report of NiV escape from mAb213 neutralization upon acquisition of a mutation at Q388R, which is within OR5 (45). Epitope mapping is useful for the rational selection of antibodies with distinct epitopes proximal to or outside the receptor-binding domain, which is relevant for studies aimed at identifying monoclonal antibody cocktails with synergistic properties for HNV neutralization (52–56). These constructs also have the potential to be utilized as immunogens to generate novel antibodies to distinct NiV regions through the use of chimeric OR or point mutant constructs. Moreover, the usage of head-stalk chimeras in sequential immunization approaches may lead to the generation of cross-reactive antibodies like vaccination strategies utilized for the influenza virus (57, 58).

In sum, the work presented here investigates the molecular determinants of EFNB3 usage, identifying single-point mutations that impair NiV usage of EFNB3 and interaction with Y120 of EFNB3 as important for usage of EFNB3. Additionally, we shed insight to regions important for EFNB2 binding by GhV and define antibody epitopes. Importantly, HNV constructs generated in this study may ultimately serve as tools to continue uncovering HNV entry biology, antibody epitopes, and immunogens to generate novel therapeutic antibodies.

## MATERIALS AND METHODS

### Generation of RBP mutants and EFNB constructs

All constructs were cloned into a pCAGGS backbone utilizing site-directed mutagenesis or overlap PCR and were sequence verified prior to use. All WT fusion glycoproteins were used for these experiments with the exception of the functionally rectified GhV-F as previously described (59). RBP constructs are all codon optimized and contain a C-terminal HA tag that is presented extracellularly. EFNB constructs encode EFNB, a GSG linker, P2A ribosomal skipping sequence, and puromycin-resistance gene.

### Maintenance and generation of cell lines

HEK293T and U87 cells were maintained in Dulbecco's modified Eagle medium (DMEM). All Cho cells were maintained in DMEM + F12 (Ham’s) media. All media were supplemented with 10% heat-inactivated fetal bovine serum. To generate cell lines stably expressing WT EFNB2, WT EFNB3, or mutant EFNB3-Y120F, constructs encoding the respective EFNB protein along with a GSG, P2A, and puromycin resistance gene were transfected into Cho-Parental cells. These were then put under 10 µg/mL of puromycin selection. Cell surface expression of the EFNBs was validated using sEPHB3-hFc (from R&D Cat. No. 5667-B3- 050) prior to freezing down low passage aliquots and using for experiments.

### Structural visualizations, interrogation, and protein modeling

PDB files 2VSM (NiV + EFNB2), 3D12 (NiV + EFNB3), 2VSK (HeV + EFNB2), and 4UF7 (GhV + EFNB2) were utilized for this study (21, 32, 41). Pymol (https://pymol.org) was utilized to generate all the structural models presented here. PDBePISA (https://www.ebi.ac.uk/msd-srv/prot_int/pistart.html) (60) was used to interrogate the contact residues between HNV-RBPs and cognate receptors. These residues were then annotated onto alignments generated using Multalin (http://multalin.toulouse.inra.fr/multalin/) (61) and ESPript (https://espript.ibcp.fr/ESPript/ESPript/) (62). COOT was utilized to mutate select residues as needed prior to visualization in pymol (63). Superimposed visualizations of structures of interest were generated using the “align” command in pymol given high-sequence similarity.

### Cell surface expression and receptor binding by flow cytometry

Cell surface expression of HA-tagged RBP was assessed by transfecting the respective WT or mutant constructs into HEK293T cells with BioT (Bioland Cat. No. B01-01). Two days post transfection, the cells were gently collected with 10 mM EDTA to avoid cleavage of the glycoprotein. Cells were then stained with a 1:2,000 dilution of anti-HA (Sigma-Aldrich H3663) to normalize for cell surface expression. For assessing receptor binding, human Fc (hFc)-tagged soluble EFNB2 (sEFNB2- hFc) from R&D (Cat. No. 7397-EB-050) or sEFNB3-hFc (Cat. No. 7924-EB-050) was used. Cell surface expression of EFNBs on newly generated cell lines was verified by seeding cells in a 12-well plate prior to collecting with 10 nM EDTA and staining with sEPHB3-hFc (R&D Cat. No 5667-B3-050). The Attune was used for all flow cytometry data acquisition, and data were analyzed using FlowJo software. For normalization of sEFNB2-Fc and sEFNB3-Fc binding to HNV-RBPs, the GMFI was first background subtracted (mock-transfected cell GMFI), then normalized to anti-HA for each soluble receptor. Constructs with a negative value or value <0.01 were given a value of 0.01 for data presentation on a log scale.

### Production of HNVpps, western blot analyses, and tittering HNVpps

Pseudotyped particles were produced as previously described. Two hundred ninety-three cells were seeded on poly-lysine coated plates 1 day prior to transfection with equal amounts of F and RBP or pCAGGS empty vector as a negative control. Eight hours post transfection, these cells were infected with VSV∆G bearing Renilla luciferase for 2 h and washed three times with Dulbecco's phosphate-buffered saline (DPBS). Media was replenished with DMEM + 10% fetal calf serum (FBS) containing a 1:20,000 dilution of an anti-VSV-G antibody 8G5F11 (Kerafast Cat. No EB0010), which reduces any possible background from residual VSV-G. Supernatants were collected and clarified 2 days post infection. In some cases, HNVpp was concentrated through on a 20% sucrose cushion and resuspended in DPBS. Samples were aliquot and stored in −80° to avoid multiple freeze-thaw cycles. Western blots were performed on concentrated pseudotyped particles by lysing particles in CA630, resuspending in SDS + beta-mercaptoethanol (BME), boiling the sample for 10 min at 95°C, then running on a 4%–15% pre-cast gel. The gel was then transferred to a polyvinylidene difluoride (PVDF) membrane and stained with the following primary and secondary antibodies. Membranes were washed with PBS containing 0.1% Tween-20. A mouse anti-VSV-M antibody (Kerafast 23H12 Cat. No. EB0012) was used as a loading control for the particles. The incorporation of HNV-RBP and HNV-F was detected with rabbit anti-HA antibody (Novus Cat. No. NB600-363) and rabbit anti-AU1 antibody (R&D systems nnb600-453), respectively. Goat anti-mouse or anti-rabbit antibodies labeled with Alexa-647 or Alexa-546 were primarily as a secondary antibody. For infection assays, HNVpps were subsequently tittered on permissive Cho-B2 and Cho-B3 cell lines. The next day, these HNVpp-infected cell lines were lysed, prepared for the Renilla luciferase (Promega Cat. No. E2820) assay, and read on the Cytation 3 (BioTek) as previously described (64).

### Use of CellProfiler for quantifying syncytia assay

CellProfiler (https://cellprofiler.org) (43, 44) was utilized to assess syncytia formation across several constructs in different cell lines. Our lab’s historical Cho-B2 and Cho-B3 cell lines and newly generated Cho-based cell lines were transfected with LifeACT-eGFP (Addgene) and pCAGG empty vector or the indicated HNV F and RBP. This transfection mix contained ~64% eGFP, 18% F, and 18% RBP. About 22–24 h post transfection, cells were stained with Hoechst (Abcam ab228551), then fixed with Paraformaldehyde (PFA) prior to capturing capture brightfield, GFP, and Hoechst images. The Celigo imager was used to capture the entire well. The same pipeline generated was used to analyze all images in an unbiased fashion. Notably, this pipeline contains a crop function to capture a large field of view (3,000 × 4,000 pixels) from the transfected well for analysis. Subsequent steps include identifying primary objects (Hoechst-stained nuclei and GFP+ objects) and masking nuclei to only include nuclei that have at least 50% overlap with GFP+ objects. The latter is a stringent threshold that results in ~20%–30% of GFP+ objects lacking a nucleus since many have nuclei at the periphery of the cells. However, this compromise was made to avoid overcounting nuclei in each GFP+ object. As a result, objects containing no nuclei were excluded from the analyses presented here, particularly when normalizing to the total number of GFP objects. Despite this exclusion, an average of >500 GFP+ objects was counted for each condition. After masking nuclei, nuclei were related to the GFP object they were contained within resulting in a “child” (nuclei) to “parent” (GFP+ object) relationship between these two objects. Several measurements were made within the pipeline and exported as a .csv file. Additionally, the outlines of GFP objects and nuclei were generated and saved to qualitatively assess the robustness of this system. This quantitative approach to screen for syncytia was validated in historical Cho-B2 and Cho-B3 cell lines that depict robust syncytia formation in Cho-B2 and Cho-B3 cells for NiV WT and impaired syncytia formation in Cho-B3 cells by NiV-OR11 and NiV-OR12 (Fig. S9).

## SUPPLEMENTAL MATERIAL

Figures S1 to S3 jvi.01838-23-s0001.pdf Fig. S1. Full sEFNB2 and sEFNB3 binding curves for control and Head-Stalk chimeras. Fig. S2. NiV-head confers EFNB3 usage to chimeric GhV-NiV-Head constructs. Fig. S3. NiV ORs and point mutants binding at 10 and 0.4 nM sEFNB2 and sEFNB3.

Figures S4 to S8 jvi.01838-23-s0002.pdf Fig. S4. Incorporation of OR1–OR12 into HNVpp. Fig. S5. Titering HNV-bearing NiV-OR mutants and point mutations. Fig. S6. GhV chimera cell surface expression and receptor binding. Fig. S7. Residues occluded on EFNB2 and EFNB3 upon interaction with HNV-RBPs. Fig. S8. Validation of newly generated cell lines.

Figure S9 jvi.01838-23-s0003.tiff Use and validation of CellProfiler for the automated quantification of HNV syncytium formation.

Figure S10 and Table S1 jvi.01838-23-s0004.pdf Figure S10. Recombinant NiV with selected mutants displays decreased EFNB3 usage in vitro. Table S1. Titers of HNVpp-bearing NiV-OR mutants and point mutations.

ASM does not own the copyrights to Supplemental Material that may be linked to, or accessed through, an article. The authors have granted ASM a non-exclusive, world-wide license to publish the Supplemental Material files. Please contact the corresponding author directly for reuse.
