# Supplementary material for: Structure-guided mutagenesis of Henipavirus receptor-binding proteins reveals molecular determinants of receptor usage and antibody-binding epitopes
Source: J Virol. 2024 Mar 1;98(3):e01838-23. doi: 10.1128/jvi.01838-23 (PMC10949843; doi:10.1128/jvi.01838-23)
Supplement: Figures S1 to S3 — Fig. S1. Full sEFNB2 and sEFNB3 binding curves for control and Head-Stalk chimeras. Fig. S2. NiV-head confers EFNB3 usage to chimeric GhV-NiV-Head constructs. Fig. S3. NiV ORs and point mutants binding at 10 and 0.4 nM sEFNB2 and sEFNB3. [file jvi.01838-23-s0001.pdf]

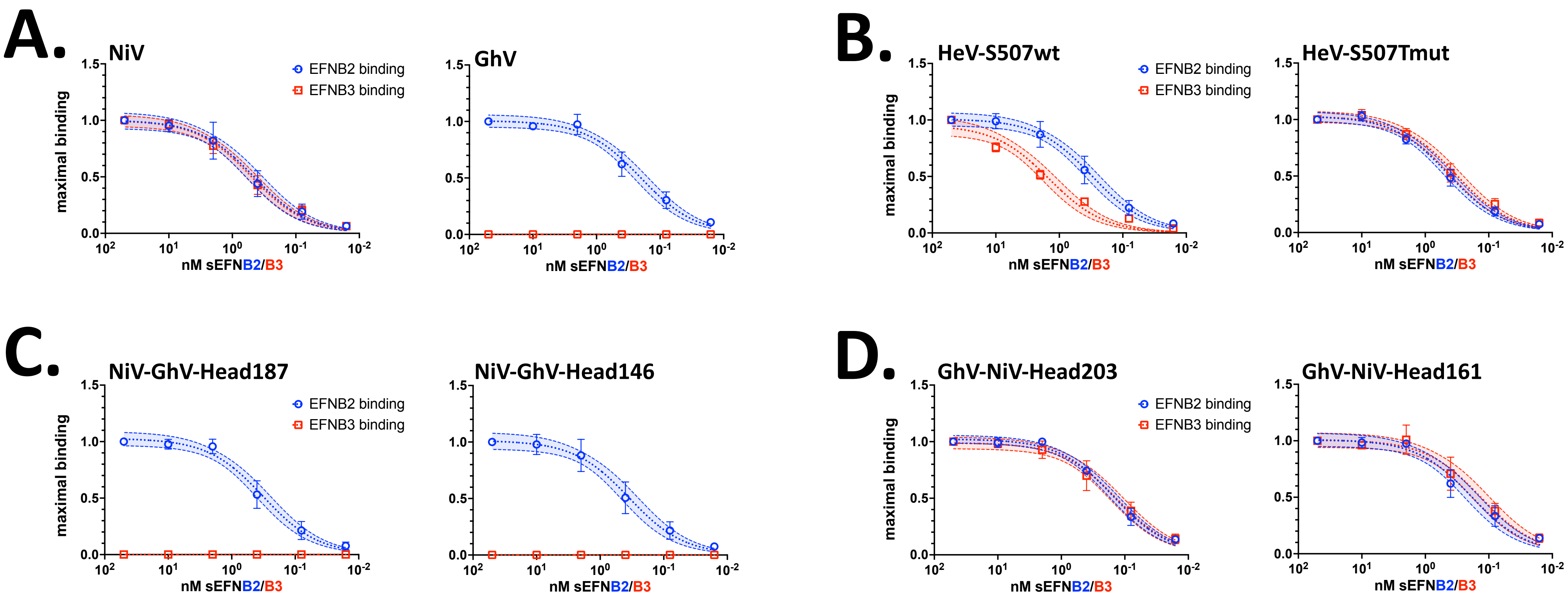

Supplemental Figure 1. Full sEFNB2 and sEFNB3 binding curves for control and Head-Stalk chimeras. Binding curves from each of the three independent experiments were used to generate individual K_ds used in **Figure 1B.** Here, the three biological replicates are aggregated into binding curves for NiV and GhV controls **(A)**, HeV constructs **(B)**, NiV-stalk, GhV-Head chimeras **(C)** and GhV-stalk, NiV-head chimeras **(D)**. For binding experiments, 293T cells were transfected with HA-tagged HNV glycoprotein then stained with a fivefold serial dilution of soluble receptor starting at 50nM and ending at 0.016nM. GMFI from binding were background subtracted, then normalized to anti-HA GMFI. This was further normalized to maximum binding at 50nM. All data points with negative GMFI after background subtraction of maximal binding <0.01 after normalizations were giving a value of 0.01 so that the point, particularly EFNB3 binding, could be visualized on the graph. Graphs show the mean with error bars representing the standard deviation. The dotted lines are from the nonlinear fit and the shaded region represents the 95% confidence bands.

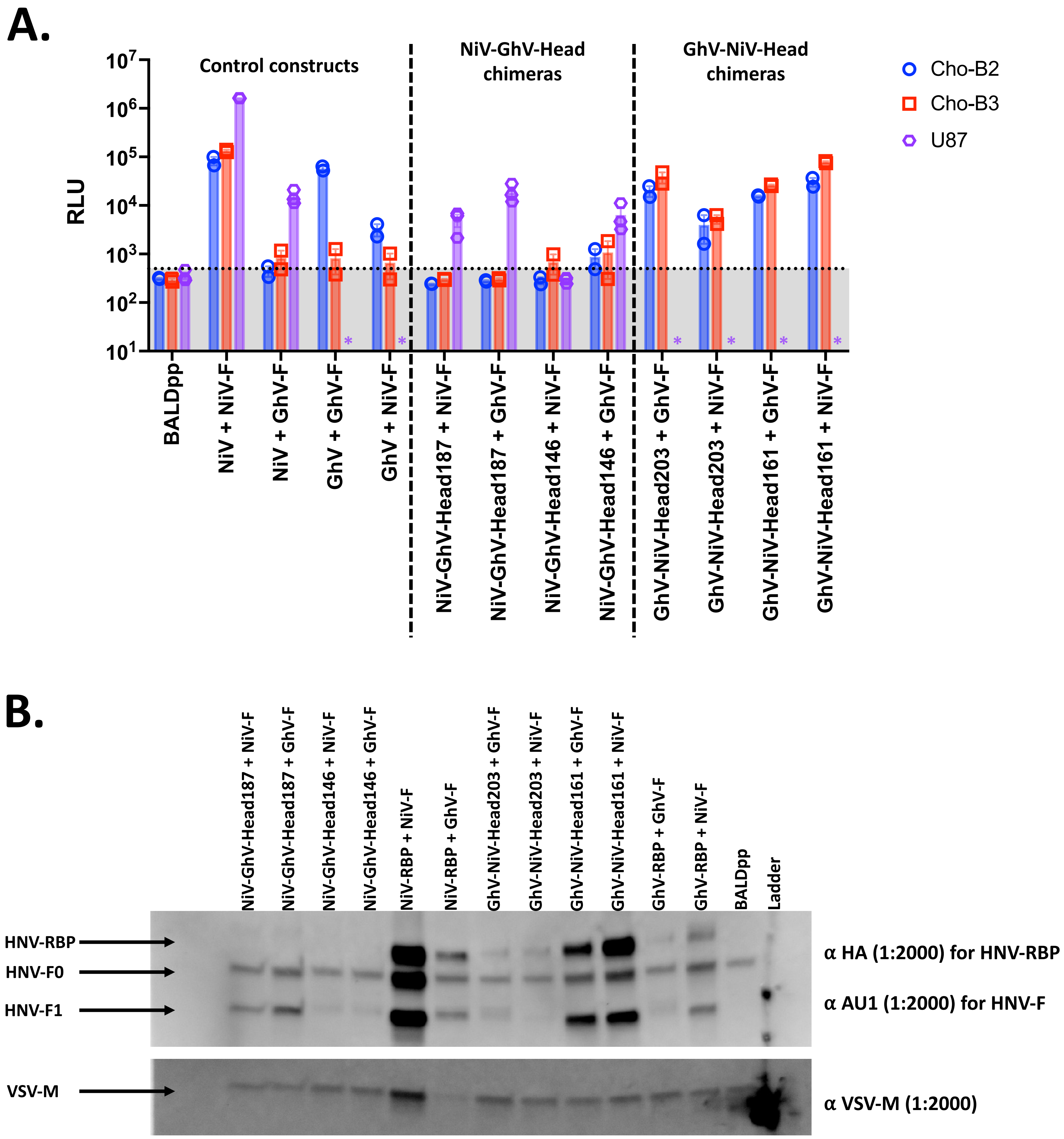

Supplemental Figure 2. NiV-head confers EFNB3 usage to chimeric GhV-NiV-Head constructs. (A) Entry of control constructs and homotypically or heterotypically complemented head-stalk chimeras into Cho-B2, Cho-B3 and U87 cells. Homotypically complemented constructs are paired with the stalk-matched F glycoprotein i.e., for NiVwt-RBP a homotypic pairing is NiV-F, and a heterotypic pairing is GhV-F. Cells were infected and processed to read Renilla luciferase generated RLUs as described in the methods. All points shown, except for homotypic NiVwt-RBP, are from a 1:10 dilution of sucrose concentrated HNVpp. Due to high titers in U87 cells, all data for NiVwt is from the 1:10,000 dilution. The dotted line at 500 RLU is indicative of the background we observe with BALDpp (particles produced with Empty vector transfected cells, then infected, and prepared as normal for all other HNVpps) in the Renilla luciferase system. As a result, values not above this threshold are interpreted as no entry in that cell line. Presented are the results from one experiment performed in technical duplicates for Cho-B2 and Cho-B3 cells and technical triplicates for U87 cells. U87 entry was not tested for conditions bearing an asterisk. **(B)** Western blot of incorporation of HNV F and RBP glycoproteins into HNV pseudotyped particles (pp). HNVpp were generated and prepared for western blotting described in the Methods. Anti-HA and anti-AU1 antibodies were used to detect HNV-RBP and F, respectively, then imaged using anti-Rabbit 647 secondary. Anti-VSV-M was utilized as a loading control and detected with an anti-Ms 546 secondary.

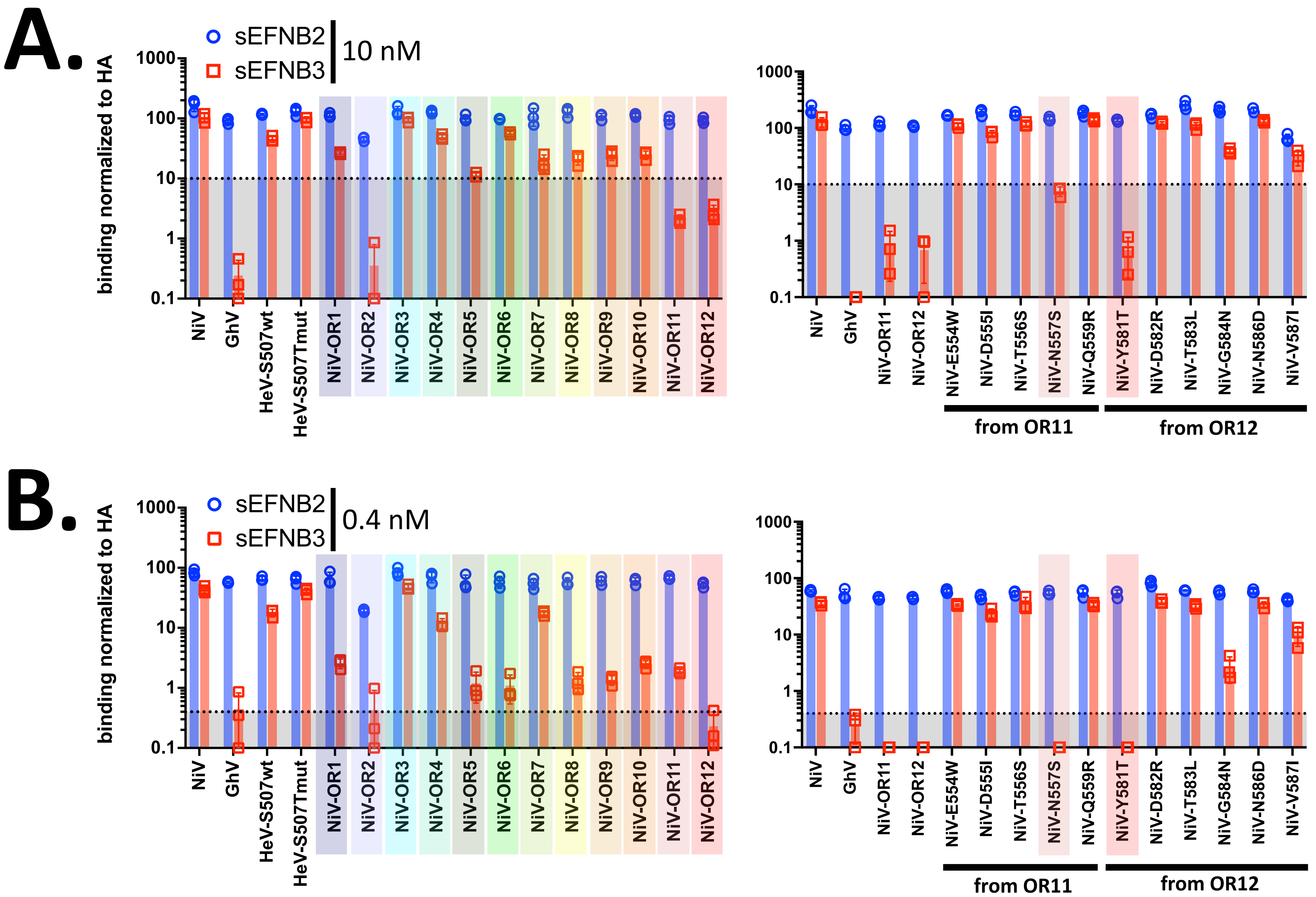

Supplemental Figure 3. NiV OR regions and point mutants binding at 10nM and 0.4nM sEFNB2 and sEFNB3. Receptor binding at 10nM **(A)** or 0.4nM **(B)** was performed and analyzed as described in **Figure 2B**. Briefly, constructs were transfected with HA-tagged RBP, then stained with Fc tagged soluble receptor. Data presented are background subtracted GMFI normalized to anti-HA and presented are the results from 3 independent biological replicates. Dotted lines are drawn at 10 **(A)** or 0.4nM **(B)** to visualize a stringent threshold for receptor binding. Bars are colored according to the coloring scheme in **Figure 2A right**.
