# Supplementary material for: Structure-guided mutagenesis of Henipavirus receptor-binding proteins reveals molecular determinants of receptor usage and antibody-binding epitopes
Source: J Virol. 2024 Mar 1;98(3):e01838-23. doi: 10.1128/jvi.01838-23 (PMC10949843; doi:10.1128/jvi.01838-23)
Supplement: Figures S4 to S8 — Fig. S4. Incorporation of OR1–OR12 into HNVpp. Fig. S5. Titering HNV-bearing NiV-OR mutants and point mutations. Fig. S6. GhV chimera cell surface expression and receptor binding. Fig. S7. Residues occluded on EFNB2 and EFNB3 upon interaction with HNV-RBPs. Fig. S8. Validation of newly generated cell lines. [file jvi.01838-23-s0002.pdf]

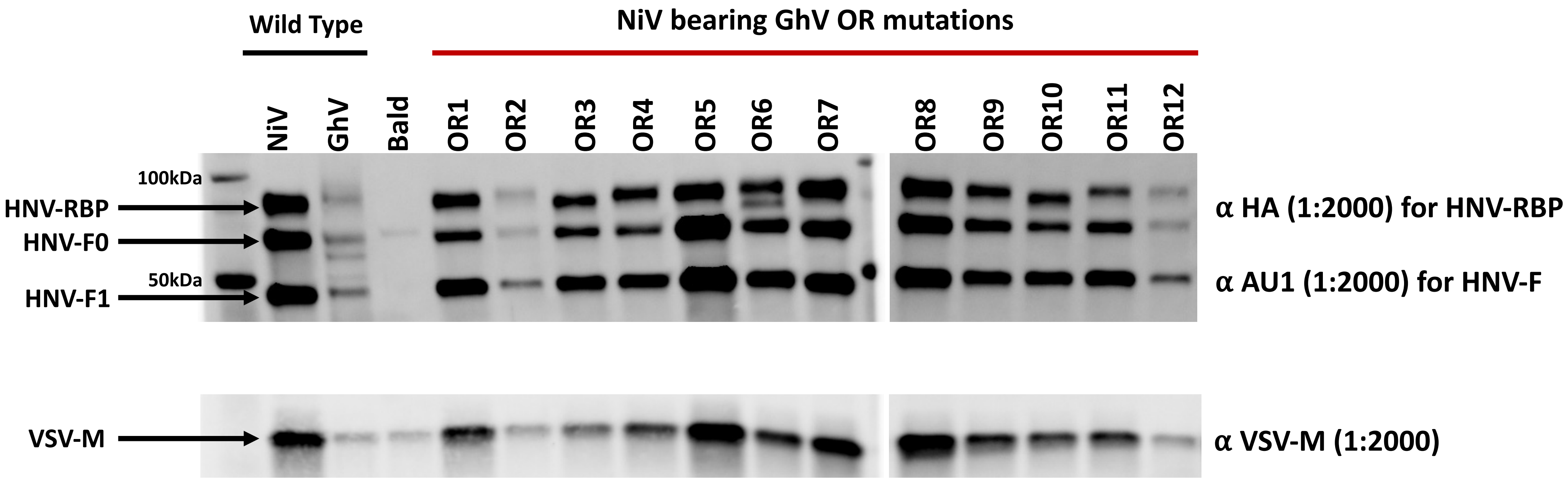

Supplemental Figure 4. Incorporation of OR1-12 into HNV pseudotyped particles (HNVpp). Incorporation of OR1-12 mutants was assessed by western blots as described in **supplemental figure 2A** with one exception. In this western blot, anti-Ms Alexa-647 secondary antibody was used due to the faint signal from the earlier use of Alexa-546 conjugated secondary. Notably, the introduction of GhV residues from OR2 (K**N**CTR) into a NiV backbone (previously bearing GSCSR at that position) introduces an N-linked glycan to NiV-OR2, which is reflected in the modest size increase relative to the other OR mutants. Additionally, GhV residues from OR10 (SEQVAE) remove an N-linked glycan from NiV (previously bearing S**N**QTAE at that position), resulting in a size decrease relative to other OR mutants.

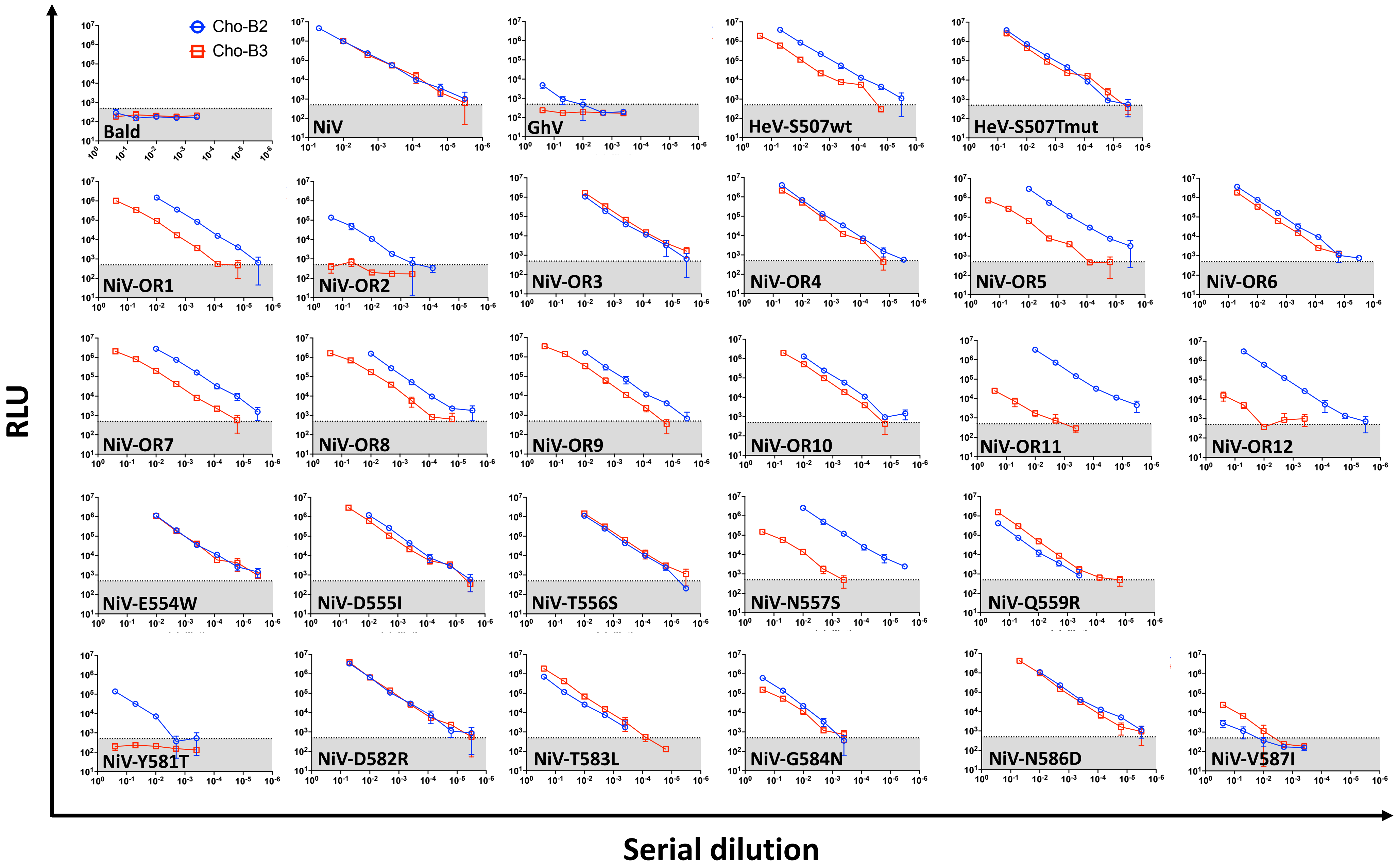

Supplemental Figure 5. Titering HNVpp bearing NiV-OR mutants and point mutations. HNVpp were prepared using the VSVΔG pseudotyping system as described in the Methods. HNVpp bearing wt or mutant RBPs and their homotypic Fusion glycoproteins were then utilized to screen for deficits in EFNB3 usage in Cho cells engineered to stably express EFNB2 (Cho-B2) or EFNB3 (Cho-B3). The dotted line at 500 RLUs indicates the background of the Renilla Luciferase assay in our system. Entry not above this threshold is interpreted as no entry. This experiment was done in technical duplicates and repeated once for a biological replicate. Shown are the results of a representative biological replicate with error bars representing the standard deviation of the mean.

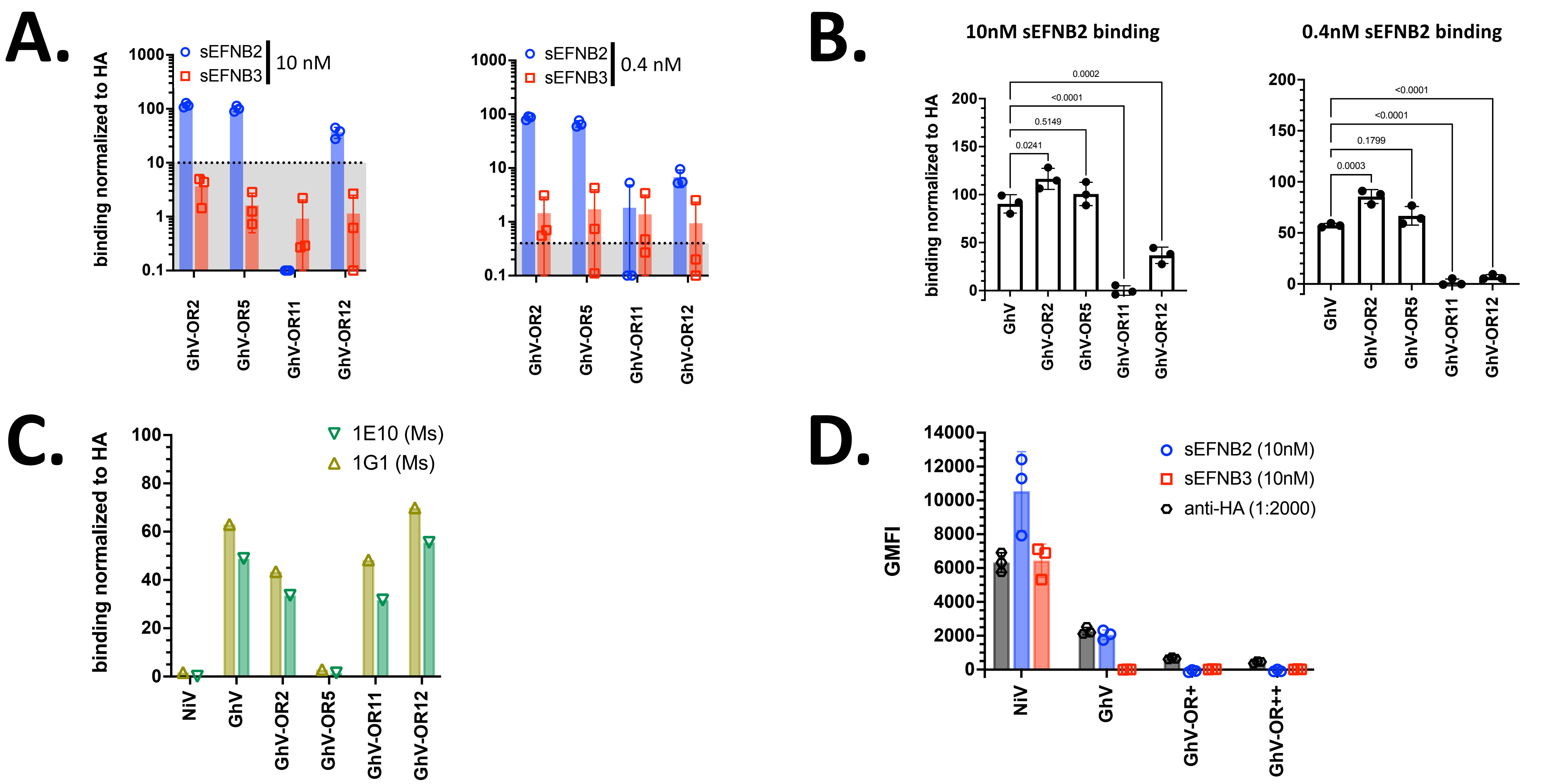

Supplemental Figure 6. GhV chimeras cell surface expression and receptor binding. (A) GhV OR2, 5, 11, and 12 binding 10 or 0.4nM EFNB2 and EFNB3. Binding was assessed as previously described in Figure 2.3 and supplemental Figure 2.3. Presented are the results of three independent, biological replicates. Dotted lines are at 10 and 0.4 for binding at 10nM and 0.4nM, respectively. **(B)** GhV binding to 10 or 0.4nM EFNB2. Binding performed and analyzed as described in Figure 2.5D. n = 3 independent biological replicates and p values are indicated on top of each comparison. Notably, OR2 showed no statistical significance at 2nM, but shows statistical significance for EFNB2 binding at 10 and 0.4nM. This suggests GhV-OR2, which removes a glycosylation site from GhV, may improve the binding affinity for EFNB2 and may ultimately improve EFNB2 usage. Additionally, OR11 and OR12 show severely impaired EFNB2 binding even at 10nM. **(C)** Assessing cell surface expression of GhV-OR with GhV-G specific monoclonal antibodies. Two mouse monoclonal antibodies previously generated by Kristopher D. Azarm, PhD in our lab were utilized for binding experiments. Experiments were performed as previously described by transfecting 293T cells, then staining 2 days post transfection with the indicated antibody. Interestingly, OR11 does not bind EFNB2, and appears to be conformationally intact at the cell surface as indicated by these two monoclonal antibodies. However, OR5 binds EFNB2 but is not bound by either antibody tested. **(D)** Cell surface expression of GhV-OR mutants bearing several OR regions from NiV. To assess whether transfer of several domains to the GhV-RBP backbone, would facilitate binding of EFNB3, we constructed GhV-OR+ and OR++ chimeras bearing NiV-OR 2+5+11+12 (OR+) or 1+2+5+7+8+11+12 (OR++). Binding was performed as previously described but presented are the background subtracted GMFI from three independent biological replicates so that the low cell surface expression of GhV-OR+ and GhV-OR++ can be fully appreciated.

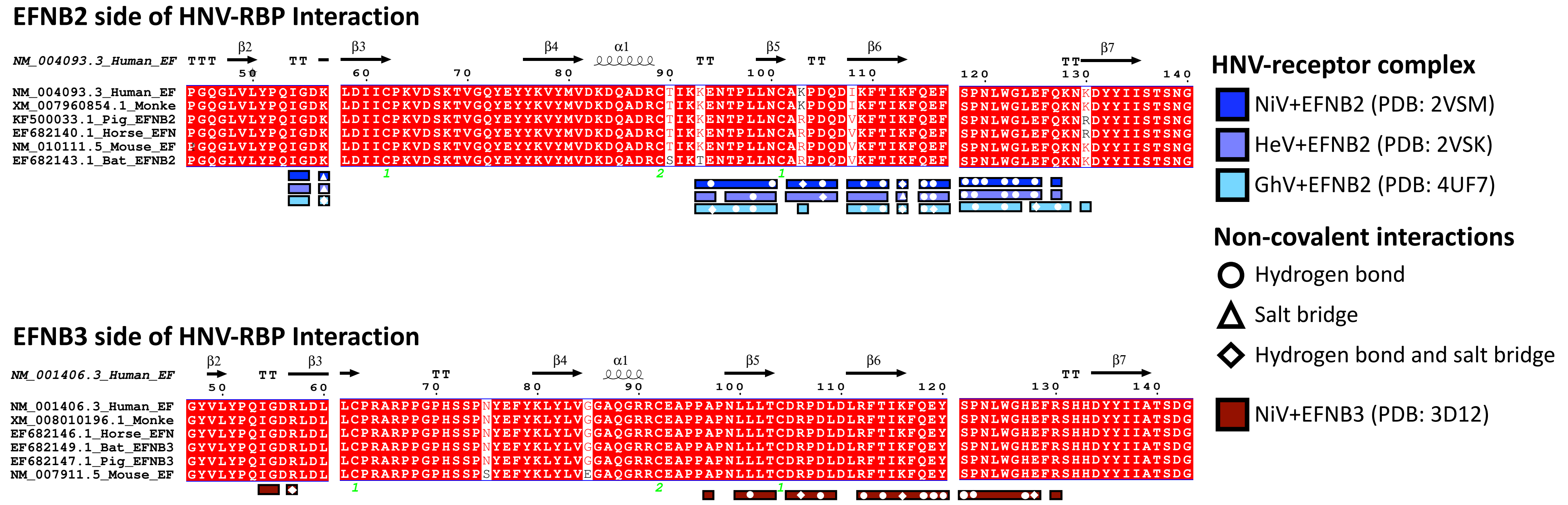

Supplemental Figure 7. Residues occluded on EFNB2 and EFNB3 upon interaction with HNV-RBPs. PDBePISA, Espript, and Multalin were used as previously described. Several representative animal species were included. Note that this is looking at the residues interacting with HNV-RBPs from the perspective of the EFNB receptors.

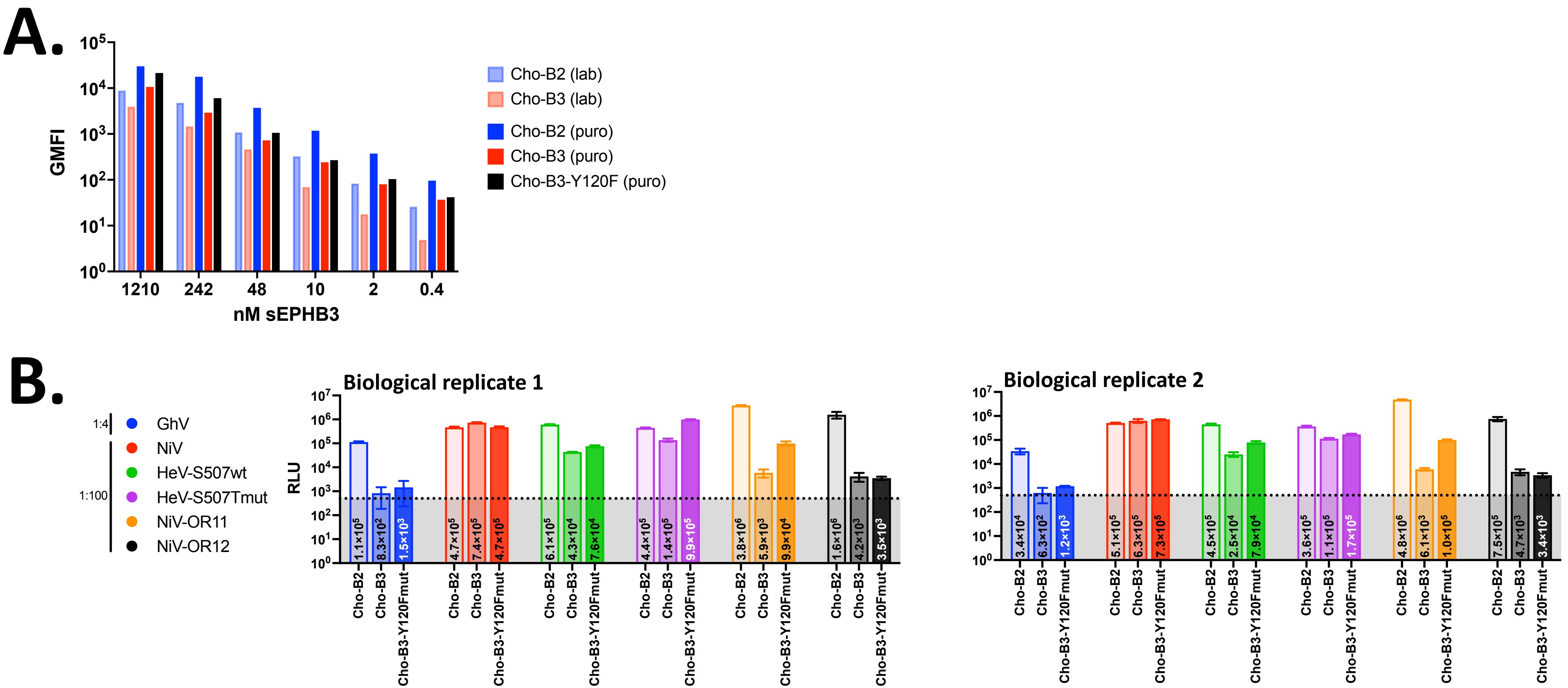

Supplemental Figure 8. Validation of newly generated cell lines. (A) Cell surface expression of EFNBs on newly generated cell lines. Presented are the background subtracted GMFIs for each of the lab's historical Cho-B2 and Cho-B3 cell lines and the newly generated, puromycin selected Cho-B2, Cho-B3, and Cho-B3-Y120F cell lines. **(B)** Raw RLUs for NiV-OR11 and OR12 entry into newly generated cell lines. Presented are the RLUs from each infection done in technical duplicates. The dotted line and corresponding shaded region at 500 indicate the background of the Renilla luciferase assay system. As expected, GhV entry into the Cho-B2 and Cho-B3-Y120F cells are at background levels for the system.
