# Supplementary figures and images for: Structure-guided mutagenesis of Henipavirus receptor-binding proteins reveals molecular determinants of receptor usage and antibody-binding epitopes
Source: J Virol. 2024 Mar 1;98(3):e01838-23. doi: 10.1128/jvi.01838-23 (PMC10949843; doi:10.1128/jvi.01838-23)

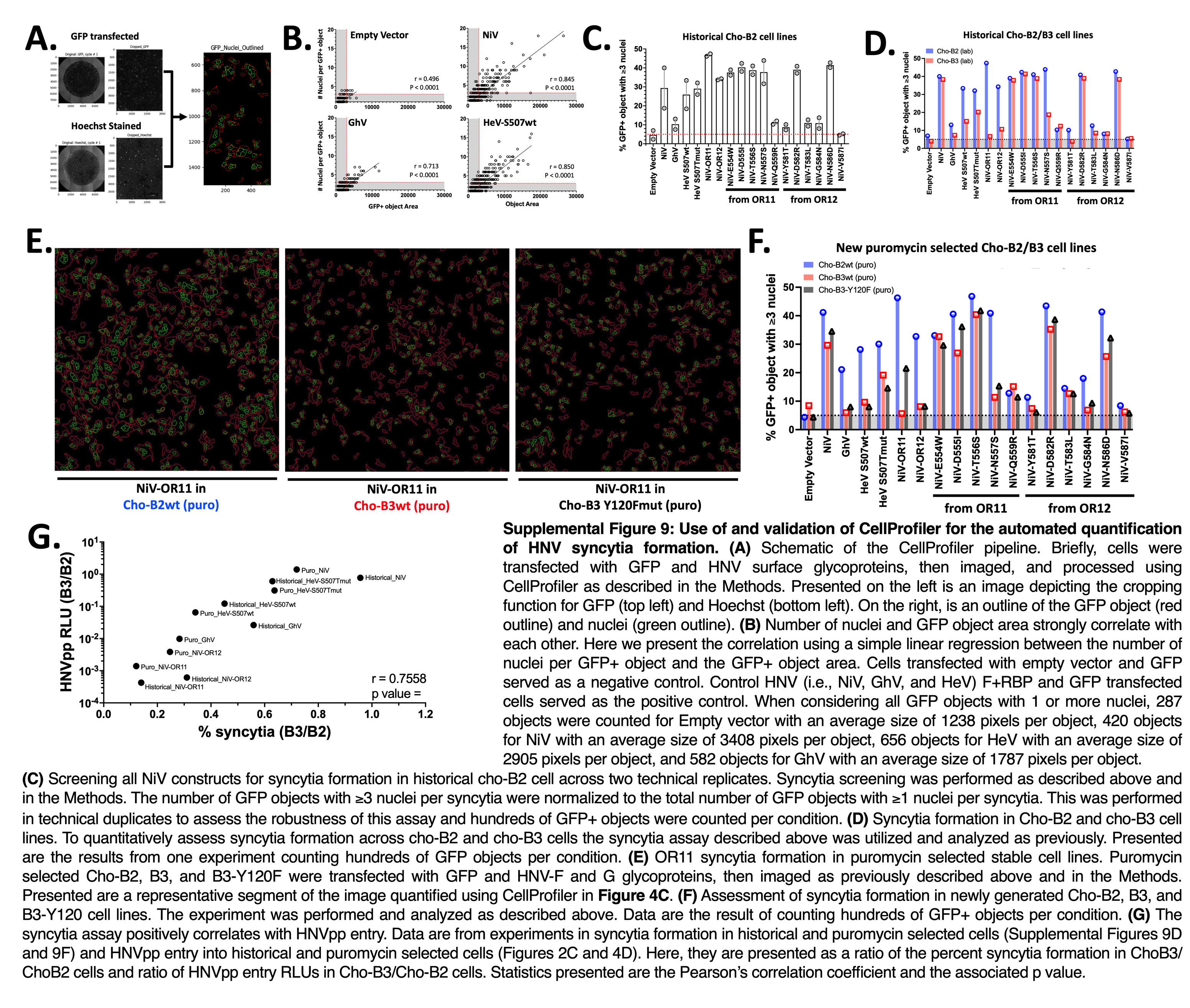

Supplement: Figure S9 — Use and validation of CellProfiler for the automated quantification of HNV syncytium formation. [file jvi.01838-23-s0003.tiff]
