# Supplementary material for: Structure-guided mutagenesis of Henipavirus receptor-binding proteins reveals molecular determinants of receptor usage and antibody-binding epitopes
Source: J Virol. 2024 Mar 1;98(3):e01838-23. doi: 10.1128/jvi.01838-23 (PMC10949843; doi:10.1128/jvi.01838-23)
Supplement: Figure S10 and Table S1 — Figure S10. Recombinant NiV with selected mutants displays decreased EFNB3 usage in vitro. Table S1. Titers of HNVpp-bearing NiV-OR mutants and point mutations. [file jvi.01838-23-s0004.pdf]

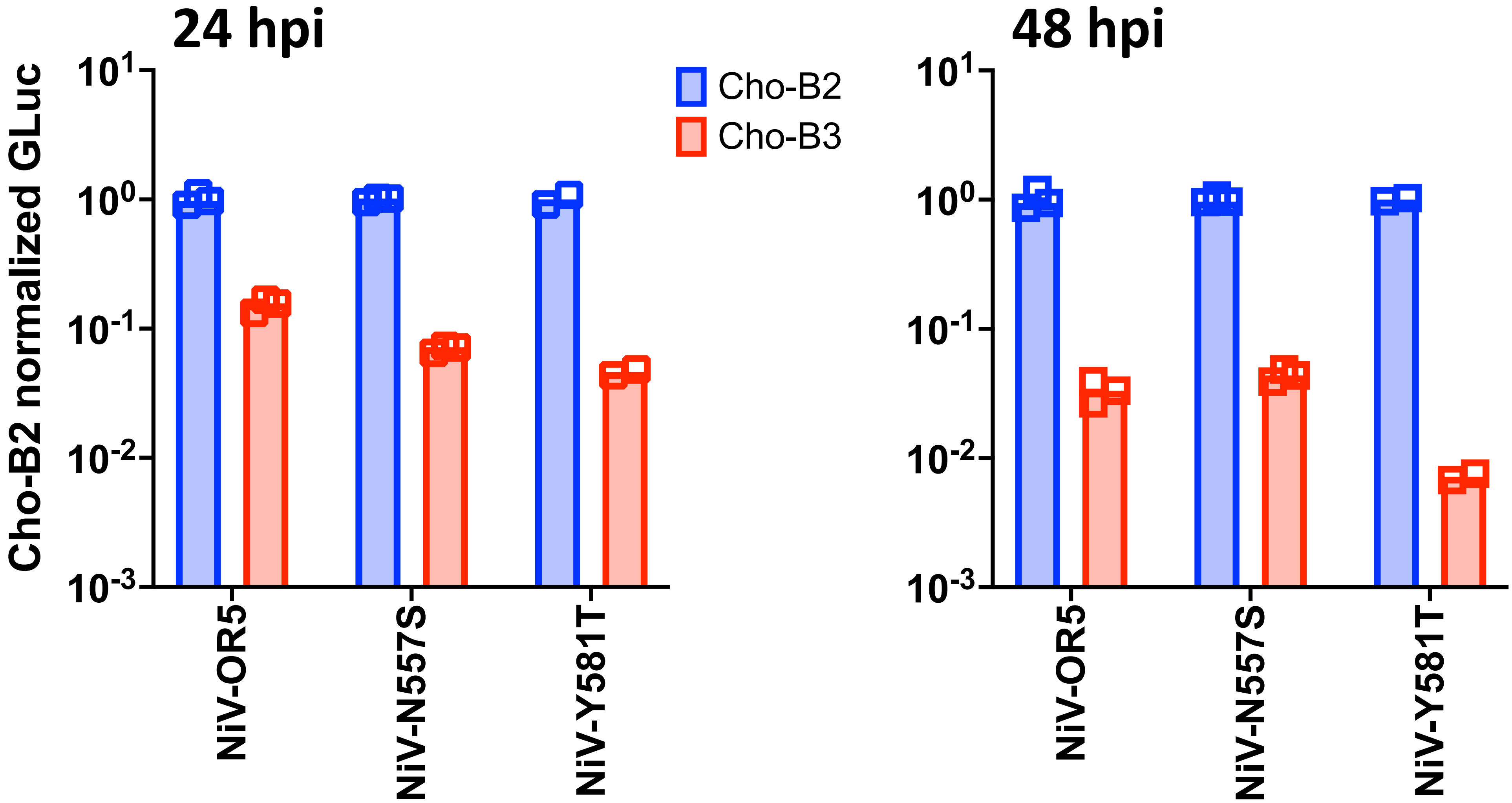

Supplemental Figure 10. Recombinant NiV with selected mutants display decreased EFNB3 usage in vitro. Briefly, recombinant Nipah virus (rNiV) bearing the indicated mutants were constructed into a GLuC-P2A-eGFP reporter virus that secretes Gaussia Luciferase. At BSL-4 laboratory facilities, these constructs were rescued on BSRT7 cells using a robust reverse genetics system[65] and amplified on Cho-B2 cells prior to freezing virus stock at -80. rNiV stock was diluted to from neat to 1/3125, then serial dilutions were used to infect Cho-B2 and Cho-B3 cells prior to collecting supernatants at 24 and 48 hours post infection (hpi). Data from the 1/625 dilution is presented above and was normalized to the Cho-B2 average for the respective sample. NiV-OR5 and NiV-N557S were performed in technical triplicates while NiV-Y581T was performed in technical duplicates.

	Construct	Avg. Cho-B2 Titer	Avg. Cho-B3 Titer	ChoB3 Titer/ChoB2 Titer
**Controls**	BALD	4.47E+00	4.47E+00	1.0000
	NiV	6.91E+06	6.91E+06	1.0000
	GhV	1.34E+03	4.47E+00	**0.0033**
	HeV-S507wt	4.16E+06	2.82E+05	0.0678
	HeV-S507Tmut	1.41E+06	1.41E+06	1.0000
**OR mutants**	NiV-OR1	6.91E+06	2.82E+05	0.0408
	NiV-OR2	1.12E+04	4.69E+01	0.0042
	NiV-OR3	6.91E+06	3.46E+06	0.5000
	NiV-OR4	4.16E+06	8.46E+05	0.2034
	NiV-OR5	6.91E+06	1.69E+05	0.0245
	NiV-OR6	4.16E+06	8.46E+05	0.2034
	NiV-OR7	6.91E+06	2.82E+05	0.0408
	NiV-OR8	4.16E+06	2.82E+05	0.0678
	NiV-OR9	6.91E+06	8.46E+05	0.1224
	NiV-OR10	4.16E+06	8.46E+05	0.2034
	NiV-OR11	6.91E+06	6.72E+03	**0.0010**
	NiV-OR12	4.16E+06	1.34E+03	**0.0003**
**OR11 point mutants**	NiV-E554W	6.91E+06	6.91E+06	1.0000
	NiV-D555I	4.16E+06	4.16E+06	1.0000
	NiV-T556S	4.16E+06	6.91E+06	1.6611
	NiV-N557S	6.91E+06	1.12E+04	**0.0016**
	NiV-Q559R	3.37E+04	1.69E+05	5.0178
**OR12 point mutants**	NiV-Y581T	1.12E+04	4.47E+00	**0.0004**
	NiV-D582R	4.16E+06	1.41E+06	0.3389
	NiV-T583L	5.62E+04	2.82E+05	5.0178
	NiV-G584N	5.62E+04	3.37E+04	0.5996
	NiV-N586D	6.91E+06	4.16E+06	0.6020
	NiV-V587I	4.46E+02	2.24E+03	5.0224

Supplemental Table 1. Titers of HNVpp bearing NiV-OR mutants and point mutations. HNVpp were prepared using the VSVΔG pseudotyping system as described in the Methods and tittered on Cho-B2 or Cho-B3 cells. This was performed in technical duplicates and repeated once for two biological replicates. The table displays the average titer from each biological replicate and a ratio of the ChoB3:ChoB2 titers.
